# *Lactobacillus reuteri*-derived HDCA suppresses PEDV replication while alleviating virus-triggered inflammation in piglets

**DOI:** 10.3389/fmicb.2025.1669658

**Published:** 2025-11-12

**Authors:** Zhibiao Bian, Qianqian Li, Hongchao Gou, Yan Li, Zhiyong Jiang, Pinpin Chu, Shaolun Zhai, Huahua Kang, Chunling Li, Guanghui Zhao

**Affiliations:** 1 College of Veterinary Medicine, Northwest A&F University, Yangling, China; 2 State Key Laboratory of Swine and Poultry Breeding Industry, Institute of Animal Health, Guangdong Academy of Agricultural Sciences, Guangzhou, China; 3 Guangdong Provincial Key Laboratory of Livestock Disease Prevention, Guangzhou, China; 4 Scientific Observation and Experiment Station of Veterinary Drugs and Diagnostic Techniques of Guangdong Province, Guangzhou, China

**Keywords:** *Lactobacillus reuteri*, HDCA, PEDV, gut microbiota, piglets

## Abstract

**Background:**

Porcine epidemic diarrhea virus (PEDV) causes severe diarrhea, vomiting, and high mortality in neonatal piglets, but no fully effective treatments or vaccines are currently available. Although gut microbiota transplantation can alleviate post infection symptoms, the specific protective bacterial strains or metabolites involved, along with their underlying mechanisms of action against PEDV, remain unclear.

**Results:**

Oral administration of *L. reuteri* GZ-1 or its metabolite hyodeoxycholic acid (HDCA) to three-day-old piglets significantly mitigated clinical symptoms and improved survival outcomes following PEDV challenge. This protection was achieved through five-day pretreatment preceding viral exposure. Both interventions substantially preserved the intestinal architecture, maintaining normal villus height and goblet cell density while markedly reducing PEDV loads in jejunal tissue. Metabolomic profiling established HDCA—a secondary bile acid derivative of *L. reuteri* metabolism—as the core protective mediator. The direct antiviral activity of HDCA against PEDV was subsequently confirmed through complementary *in vitro* and *in vivo* experimental validation. Integrated transcriptomic and proteomic analyses revealed a dual mechanistic pathway underlying HDCA efficacy: (1) suppression of NF-κB-driven inflammatory cascades and (2) activation of interferon-stimulated gene 15 (ISG15)-dependent antiviral pathways.

**Conclusion:**

This study establishes the *L. reuteri*-HDCA-TGR5-IFNβ-ISG15 metabolic axis as a novel antiviral pathway. This study identified microbial-derived HDCA as a key effector metabolite that mediates protection against PEDV through the coordinated suppression of inflammation and enhancement of antiviral defenses. These findings highlight microbial-metabolic crosstalk as a promising therapeutic strategy against enteric coronaviruses and provide foundational evidence for commensal-derived interventions to manage porcine epidemic diarrhea.

## Introduction

PEDV is an extremely contagious coronavirus that not only infects its primary host, pigs but also has the potential to cross species barriers and is capable of infecting cells from a variety of mammals, including humans (Niu et al., 2023; Lv et al., 2022). PEDV spreads mainly through fecal–oral contact and airborne transmission, targeting and invading small intestinal epithelial cells for replication and dissemination (Li et al., 2018). This process directly disrupts intestinal barrier function, triggering severe inflammatory responses such as diarrhea and vomiting, making PEDV a major pathogen responsible for high morbidity and mortality rates in pig populations (Zhai et al., 2023). The critical lack of effective antiviral drugs or effective vaccines against PEDV has led to mortality rates approaching 100% in neonatal piglets, thus triggering substantial scientific and veterinary concern regarding its pandemic potential and economic impact (Beall et al., 2016; Liang et al., 2024). In recent years, frequent mutations in PEDV strains have posed substantial challenges to the efficacy of existing vaccines, including issues such as immune escape due to antigenic variation and insufficient cross-protection between different genotypes (Yang et al., 2024; Xu et al., 2023). Consequently, the development of novel and effective prevention and control strategies has become a critical focus of current research. Studies have shown that transplanting feces from healthy adult pigs to piglets can increase the survival rate of piglets infected with PEDV by regulating the homeostasis of the gut microbiota (Xing et al., 2024). These findings indicate that the gut microbiota is essential for PEDV prevention and management.

With the development of multiomics sequencing technology, the influence and regulation of microorganisms on the host have been gradually revealed. In particular, the effects of commensals on the treatment and prevention of intestinal diseases have received extensive attention. Commensals not only maintain intestinal health by modulating the composition of the gut microbiota but also combat pathogenic invasions through the transformation and synthesis of their metabolites (Zhou et al., 2021; Segrist and Cherry, 2020; Antunes et al., 2019; Wypych et al., 2019; Kim et al., 2020). Emerging evidence highlights the crucial role of secondary bile acids, microbial-derived metabolites, in regulating host pathogenesis by activating immune responses and preserving intestinal structural integrity. However, current research predominantly focuses on host–microbe interactions while overlooking the direct mechanistic interplay between the gut microbiota/metabolites and the pathogens themselves (Xing et al., 2024; Feng et al., 2015; Grau et al., 2020; Sun et al., 2023; Xie et al., 2023).

We postulate that the gut microbiota and its metabolism may inhibit PEDV replication. To test this hypothesis, we employed 16S rRNA gene amplicon and metagenomic sequencing to identify core microbial taxa associated with PEDV resistance. These core strains were subsequently transplanted into a PEDV-challenged model. Multiomics approaches—including targeted/untargeted metabolomics, transcriptomic profiling, and proteomic analysis—were systematically integrated to delineate the antiviral effects of these core commensals and their metabolites. This comprehensive strategy aims to elucidate the mechanistic interplay between gut-derived microbes and their metabolic repertoire in suppressing PEDV pathogenesis.

## Materials and methods

### PEDV, *Lactobacillus reuteri* and cells

The PEDV strain GDqy2017 was isolated in 2017 and stored in the laboratory. *L. reuteri* GZ-1 was isolated in 2022 via De Man, Rogosa and Sharpe (MRS) medium at 37 °C under anaerobic conditions. Long Line Continuous - Pig Kidney 1 (LLC-PK1) and Intestinal Porcine Epithelial Cell - Jejunum 2 (IPEC-J2) cells (ATCC) were maintained in Dulbecco’s Modified Eagle Medium/Nutrient Mixture F-12 (DMEM/F12) or Minimum Essential Medium‌ (MEM) supplemented with 10% heat-inactivated FBS (Gibco).

### Animals and experimental design

Design 1: Investigation of gut microbiota differences between healthy and PEDV-infected piglets. This experiment aimed to compare the gut microbiota of healthy and PEDV-infected piglets. To this end, seven-day-old Landrace piglets were screened to ensure they were negative for PEDV and Porcine Reproductive and Respiratory Syndrome Virus (PRRSV) antibodies in serum, and negative for PEDV, rotavirus, porcine deltacoronavirus, PRRSV, pseudorabies virus (PRV), classical swine fever virus (CSFV), porcine circovirus type 2 (PCV2), and porcine circovirus type 3 (PCV3) in feces. Using the qualified piglets (*n* = 10), a PEDV challenge was performed (1,000 TCID_50_), while healthy controls (*n* = 9) were established. Postmortem jejunal contents were then collected for 16S rRNA gene amplicon sequencing and metagenomic sequencing to analyze microbial differences.

Design 2: Evaluation of the protective effect of *L. reuteri* against PEDV infection. This experiment was designed to investigate whether intestinal colonization with *L. reuteri* confers protection against PEDV infection. Landrace sows were screened to ensure their colostrum was negative for PEDV and PRRSV antibodies and their feces were negative for PEDV, rotavirus, porcine deltacoronavirus, PRRSV, PRV, CSFV, PCV2, and PCV3. Piglets from qualified sows were fed colostrum until 3 days of age. They were then orally administered either live (LLR) or killed (KLR) *L. reuteri* (1 × 10^9^ CFU/day/piglet), while the control group received MRS medium, with five piglets per group. A PEDV challenge was conducted on day 5 post-intervention to monitor diarrhea status and mortality rates to determine the protective effect of *L. reuteri*.

Design 3: Evaluation of the protective effects of *L. reuteri* and HDCA against PEDV infection. This experiment aimed to investigate the protective effects of *L. reuteri* and HDCA against PEDV infection in piglets. To ensure a pathogen-free baseline, Landrace sows were screened for the absence of PEDV in colostrum, the absence of PEDV and PRRSV antibodies in serum, and the absence of PEDV, rotavirus, porcine deltacoronavirus, PRRSV, PRV, CSFV, PCV2, and PCV3 in feces. All sows and their neonatal offspring were confirmed seronegative for PEDV and PRRSV. Piglets were fed colostrum until 3 days of age and then orally administered live *L. reuteri* (LLR; 1 × 10^9^ CFU/day/piglet), HDCA (10 mg/kg/day), or Dimethyl sulfoxide (DMSO) as the vehicle control, with five piglets per group. Safety assessments and PEDV challenges were conducted on day 5 post-intervention to monitor diarrhea status and mortality rates, thereby evaluating the protective efficacy of *L. reuteri* and HDCA.

### Humane endpoints and euthanasia

Piglets exhibiting severe clinical signs (watery diarrhea, inability to stand, or dyspnea) were euthanized immediately to preclude suffering. Euthanasia was performed via intravenous injection of pentobarbital sodium salt (100 mg/kg) into the marginal ear vein. Death was confirmed by absence of corneal reflex and cessation of cardiac activity for >5 min. No anesthesia was administered, as all sampling procedures were conducted postmortem. All procedures adhered to AVMA Guidelines for the Euthanasia of Animals (2020).

### *In vitro* treatment strategies with HDCA

At 80% confluence, IPEC-J2 monolayers were supplemented with 100 μM HDCA, 20 μM TGR5 antagonist (SBI-115), 20 μM TGR5 Receptor Agonist (CCDC), or DMSO (the culture medium for the mock and PEDV groups was replaced with basal DMEM). After 2 h of incubation, the cells were harvested for measurement of G Protein-Coupled Bile Acid Receptor 1 (TGR5).

In a parallel experiment, after 2 h of incubation, the medium from each well was aspirated and stored for later use. All the wells (except those in the mock group) were inoculated with PEDV at a multiplicity of infection (MOI) of 0.1 for 2 h. The supernatant was then discarded, and the cells were replenished with the previously collected conditioned medium to maintain the original treatment environment, with fresh bovine trypsin added to a final concentration of 3.75 μg/mL. After 12 h, cell samples were obtained to assess the Interferon-β (IFN-β) and Interferon-Stimulated Gene 15‌ (ISG15) levels. In another parallel experiment, after 24 h of incubation, the supernatants were collected for PEDV copy number and titre determination, while the cells were harvested for western blot analysis of PEDV-N. All treatments included triplicate wells, and experiments were repeated independently three times.

### Measurement of intracellular cAMP levels by ELISA

IPEC-J2 cells were counted and seeded at 1 × 10^6^ cells per well. After washing with PBS to remove impurities, cells were suspended in DMEM supplemented with 100 μM HDCA, 20 μM SBI-115, 20 μM CCDC, or DMSO (control groups received basal DMEM). Following 1.5 h incubation, all cells were collected into EP tubes and subjected to three freeze–thaw cycles at −80 °C to fully release intracellular Cyclic Adenosine Monophosphate (cAMP). After centrifugation at 1,500 × g for 10 min, supernatants were collected. cAMP concentrations were measured using a commercial ELISA kit (Elabscience, Cat# E-EL-0056) strictly following the manufacturer’s instructions.

### Quantification of PEDV titers by indirect immunofluorescence assay

PEDV titres were determined via indirect immunofluorescence assay (IFA). Briefly, following standard processing (4% PFA fixation, 0.1% Triton X-100 permeabilization), the cells were blocked with 5% skim milk (1 h). After primary antibody treatment (1:500, 4 °C overnight), the samples were incubated with a FITC-conjugated secondary antibody (1:500) for 1 h at RT. Nuclei were counterstained with DAPI (5 min). Images were captured with a bioimaging instrument (EVOS FL AUTO, China).

### *Lactobacillus reuteri* bile acid metabolism *in vitro*

Upon reaching an OD600 of 0.3, *L. reuteri* cultures were treated with 100 μM Taurocholic Acid (TCA), Hyocholic Acid (HCA), or Taurochenodeoxycholic Acid (TCDCA). For the bile salt hydrolase (BSH) inhibition assays, additional wells were treated with 100 μM HCA + Gut restricted-7 (GR7). Both the negative control and MRS medium control groups were included. Cultures were terminated at 72 h by flash-freezing in liquid nitrogen.

### Western blot analysis

The cells were harvested and lysed in RIPA buffer (containing PMSF) on ice for 30 min. The mixture was subsequently centrifuged (12,000 × g, 15 min, 4 °C) to collect the supernatants. Protein concentrations were measured with a BCA assay (Vazyme, China). Equal quantities of protein were resolved by 10% SDS–PAGE and subsequently transferred to PVDF membranes (Millipore). The membranes were blocked with 5% nonfat milk (BD Difco, USA) in TBST for 1 h and then incubated overnight at 4 °C with primary antibodies.: PEDV-N (1:2000), GAPDH (1:2000), and ISG15 (1:2000). After being washed with TBST (3 cycles), the membranes were incubated with HRP-conjugated secondary antibodies (1:10000) for 1 h. Protein signals were detected via a chemiluminescence analysis system (Tanon, China). Band intensities were normalized relative to those of GAPDH.

### Histological analysis

After fixation, the jejunum was embedded in paraffin and sectioned. The sections were pretreated and stained with hematoxylin and eosin (H&E) for histological analysis. Additionally, Alcian blue-periodic acid Schiff (AB-PAS) staining was performed via a commercial kit (Servicebio, China). Following dehydration, tissue sections were coverslipped and subsequently imaged using a PANNORAMIC digital slide scanner (3DHISTECH, Hungary). Goblet cell counts and villus height-to-crypt depth ratios were quantified using CaseViewer 2.4 (3DHISTECH, Hungary) and Image-Pro Plus 6.0 software (Media Cybernetics, USA).

For immunofluorescence staining of PEDV in the jejunum, tissue sections were blocked with 3% bovine serum albumin (BSA), followed by sequential incubation with PEDV-N primary antibodies and FITC-conjugated secondary antibodies (Servicebio, China). Following counterstaining with DAPI and quenching of tissue autofluorescence, the sections were mounted for microscopic imaging. The percentage of positive cells (number of positive cells / total number of cells) was statistically analyzed from images captured at 20x magnification using Aipathwell software.

### 16S rRNA gene amplicon sequencing and data analysis

The jejunal contents were collected and subjected to genomic DNA extraction via the E.Z.N.A.® system (Omega Biotek, USA) with strict adherence to the provided protocol. The extracted nucleic acids were subjected to DNase treatment and reverse transcription. The primers 338F/806R (Supplementary Table 1) amplified the V3–V4 regions of the bacterial 16S rRNA gene amplicon via PCR. The PCR products were purified through gel electrophoresis extraction and quantified via a Quantus™ Fluorometer (Promega, USA). The purified amplicons were normalized to equimolar concentrations and subjected to paired-end sequencing on an Illumina PE300 platform (Illumina, San Diego, USA). The raw sequencing data were processed through quality filtering (removal of reads with average Phred scores <20), paired-end assembly, and orientation adjustment via the Majorbio Cloud Platform. OTUs were generated from quality-filtered sequences with 97% identity. Functional prediction analysis was performed via PICRUSt2. Subsequent statistical analyses and visualizations were conducted with the Mothur v1.30.1 and Vegan v2.5 packages in the R environment.

### Measurement of cell viability using CCK-8 assay

Cells were seeded in a 96-well plate at a density of 5 × 10^3^ cells/well and incubated for 24 h. Following treatment with serially diluted test samples in DMEM for 24 h in a CO₂ incubator, the medium was aspirated and cells were washed twice with PBS. Subsequently, 110 μL of fresh DMEM containing 10 μL CCK-8 solution was added to each well. After incubation at 37 °C for 1 h, absorbance was measured at 450 nm using a microplate reader. Cell viability was calculated as: Viability (%) = [(As − Ab) / (Ac − Ab)] × 100, where As = test well (cells + treatment + CCK-8), Ac = control well (untreated cells + CCK-8), and Ab = blank well (medium + CCK-8 only). All tests included triplicate wells.

### Detection of BSH gene by PCR

Genomic DNA was extracted from bacterial cultures using a Bacterial DNA Kit (Omega Bio-tek). PCR amplification of the BSH gene was performed using the primer pair BSH-F and BSH-R (Supplementary Table 1). Each 25 μL reaction contained approximately 50 ng of template DNA, 0.5 μM of each primer, 2 × Taq Plus Master Mix II (Vazyme). Amplification was carried out in a thermal cycler under the following conditions: initial denaturation at 95 °C for 5 min; 35 cycles of denaturation at 95 °C for 30 s, primer annealing at 57 °C for 30 s, and extension at 72 °C for 45 s; followed by a final extension at 72 °C for 10 min. PCR products were analyzed by electrophoresis on a 1% agarose gel stained with Super GelBlue, alongside a DNA molecular weight marker. Negative controls (MRS medium) were included in each run.

### Quantitative PCR (q-PCR)

Total RNA was extracted from the jejunum using a Total RNA Kit (Omega Biotek, USA) according to the manufacturer’s instructions. PEDV RNA was isolated from cell culture supernatants via a Viral RNA Kit (Omega Biotek, USA). After DNase treatment and cDNA synthesis, SYBR Green or TaqMan probe qPCR was performed. PEDV load, key bile acid metabolism regulators, and TGR5 and IFN-*β* mRNA levels were quantified via SYBR Green assays. Relative gene expression was calculated via the 2^-ΔΔCt^ method, with GAPDH used as the reference gene. For absolute quantification of PEDV RNA in the cell culture supernatants, TaqMan probe-based qPCR (Vazyme, China) was employed. Viral copy numbers were determined via a standard curve generated from serial dilutions of a PEDV-specific recombinant plasmid.

### Total cholesterol measurement in blood and liver tissues

Total cholesterol levels in blood and liver tissues were quantified using the Beyotime Amplex Red Cholesterol Assay Kit (S0211S). Liver samples (20 mg) were homogenized in 200 μL BeyoLysis™ Buffer A for Metabolic Assay per 20 mg tissue, followed by centrifugation at 12,000 × g for 5 min at 4 °C. The supernatant was collected for analysis. A 5-fold diluted sample (50 μL) was combined with 50 μL Total Cholesterol Working Solution (containing cholesterol esterase, enzyme mix, and Amplex Red) in 96-well plates. After incubation at 37 °C for 30 min, absorbance was measured at 570 nm. Cholesterol concentrations were calculated against a 0–500 μM standard curve according to the manufacturer’s protocol.

### Metagenomic sequencing and bioinformatics analysis

Genomic DNA was extracted via the Mag-Bind® system DNA Kit (Omega Biotek, USA), followed by quality assessment (1% agarose gel electrophoresis) and fragmentation (Covaris M220; 400 bp target size). PE libraries were constructed via NEXTFLEX Rapid DNA-Seq (Bioo Scientific, USA), which involves adapter ligation, magnetic bead purification, PCR enrichment, and final library validation. The Illumina NovaSeq platform was used for sequencing. Quality-filtered reads (fastp v0.20.0: removal of adapters, reads <50 bp, Phred score <20) were *de novo* assembled (MEGAHIT v1.1.2; contigs ≥300 bp). Open reading frames (ORFs) were predicted via MetaGene (length ≥100 bp). A nonredundant gene catalogue was generated via CD-HIT (v4.6.1; 90% identity, 90% coverage). Gene relative abundance was quantified via SOAPaligner (v2.21; 95% identity). The functional annotation employed Diamond (v0.8.35; BLASTP, e value ≤1e−5) against NR (taxonomic profiling), eggNOG (COG functions), and KEGG (pathways, enzymes). CAZy annotations utilized hmmscan (CAZy database; e value ≤1e−5), while virulence factors were identified via VFDB (core and predicted databases). Carbohydrate-active enzymes and virulence factors were quantified on the basis of gene relative abundance.

### Untargeted metabolomics of jujube content via UPLC–MS/MS

Metabolites were extracted by mixing 100 μL samples with 400 μL acetonitrile:methanol (1:1, v/v) supplemented with 0.02 mg/mL L-2-chlorophenylalanine. After vortexing (30 s) and low-temperature sonication (5 °C, 40 kHz, 30 min), the proteins were precipitated at −20 °C for 30 min. The supernatant was collected by centrifugation (13,000 × g, 15 min, 4 °C), dried under nitrogen, and reconstituted in acetonitrile: water (1:1), followed by repeated sonication and centrifugation. Quality control (QC) samples, prepared by pooling equal volumes of all samples, were analysed every 5–15 injections to monitor system stability. LC–MS/MS analysis was conducted via a Thermo UHPLC-Q Exactive HF-X system with an ACQUITY HSS T3 column (100 × 2.1 mm, 1.8 μm). The mobile phases included 0.1% formic acid in water: acetonitrile (A) and acetonitrile: isopropanol (B), with gradient elution (positive/negative ion modes specified). MS parameters: ESI source (±3,500 V), 425 °C source temperature, 50 arb sheath gas, full MS resolution 60,000, and MS/MS resolution 7,500 (70–1,050 m/z range). The raw data were processed via Progenesis QI, filtered (with >80% metabolic features retained in any group), normalized, and QC filtered (RSD < 30%). Differentially abundant metabolites (VIP > 1, *p* < 0.05 via OPLS-DA and t tests) were annotated via the HMDB, Metlin, and KEGG databases. Pathway enrichment was analysed via Python scipy. Stats.

### Targeted bile acid metabolomics of jujube content via UPLC–MS/MS

Lyophilized solid samples (50 mg) were homogenized with a 6-mm grinding bead using a Wonbio-96c cryogenic grinder (−10 °C, 50 Hz, 6 min) in 400 μL of methanol:water (4:1, v/v) containing 0.02 mg/mL L-2-chlorophenylalanine. After low-temperature sonication (5 °C, 40 kHz, 30 min) and protein precipitation (−20 °C, 30 min), following centrifugation (12,000 × g, 20 min, 4 °C), the supernatants were analysed via LC–MS/MS. Quality control (QC) samples, prepared by pooling equal volumes of all extracts, were injected every 5–15 runs to monitor system stability. Chromatographic separation was performed on a Thermo UHPLC-Q Exactive HF-X system (ACQUITY HSS T3 column: 100 × 2.1 mm, 1.8 μm) with mobile phases A (0.1% formic acid in water: acetonitrile, 95:5) and B (0.1% formic acid in acetonitrile: isopropanol: water, 47.5:47.5:5). Gradient elution profiles for positive/negative ion modes were specified. The MS parameters included the ESI source (±3,500 V), 425 °C source temperature, sheath/aux gas flow (50/13 arb), 325 °C ion transfer tube, full MS/MS resolution (60,000/7,500), and DDA acquisition (70–1,050 m/z). The raw data were processed via Progenesis QI for peak alignment, matrix generation, and metabolite annotation (HMDB, Metlin, and Majorbio databases). The data matrices were filtered (>80% nonzero values in any group), normalized (sum normalization), and QC filtered (RSD < 30%). Differentially abundant metabolites (VIP > 1, *p* < 0.05 via OPLS-DA and t tests) were identified via the R package ropls (v1.6.2). Pathway enrichment analysis was conducted via Kyoto Encyclopedia of Genes and Genomes (KEGG) and Fisher’s exact tests (scipy. stats).

### Proteomic sequencing

Total proteins were extracted from samples via urea lysis buffer (8 M urea, 1% SDS) supplemented with protease inhibitors, followed by homogenization, ice lysis, and centrifugation (12,000 × g, 30 min, 4 °C). The protein concentration was determined via a BCA assay. For digestion, 100 μg of protein was reduced (10 mM TCEP, 37 °C, 60 min), alkylated (40 mM IAM, RT, 40 min), acetone-precipitated, and digested with trypsin (1:50, 37 °C, overnight). The peptides were labelled with TMT reagents, quenched with hydroxylamine, pooled, and dried. Fractionation was performed via high-pH RPLC (C18 column, 48-min gradient), and the collected fractions were concentrated. LC–MS/MS analysis used an Easy-nLC 1,200 coupled to a Q Exactive HF-X, with peptides separated on a C18 column (90-min gradient) and analysed in DDA mode (120 K MS, 45 K MS/MS). RAW data were processed via Proteome Discoverer (*p* ≤ 0.05), and the differentially expressed proteins (fold change >1.2 or <0.83, *p* < 0.05) were annotated via GO/KEGG and analysed for protein–protein interactions (STRING v10.5).

### Transcriptomic sequencing

Total RNA was extracted from tissues via TRIzol® Reagent, and its quality was assessed via a bioanalyzer (RI*N* ≥ 6.5, 28S:18S ≥ 1.0) and quantified via a NanoDrop (OD260/280 = 2.0). High-quality RNA (>1 μg) was used for Illumina® Stranded mRNA library preparation. Poly(A)-enriched mRNA was fragmented, reverse-transcribed into cDNA (SuperScript kit), and processed through end repair, adenylation, and adapter ligation. Libraries (300 bp inserts) were PCR-amplified (15 cycles), quantified (Qubit 4.0), and sequenced on a NovaSeq 6,000 (2 × 150 bp). The raw reads were trimmed (fastp) and aligned to the reference genome (HISAT2), followed by transcript assembly (StringTie). Gene expression was quantified (TPM, RSEM), and differential expression analysis was performed (DESeq2: |log2FC| ≥ 0.585, *p* ≤ 0.05). The functional enrichment of the DEGs was analysed via the GO (goatools) and KEGG (KOBAS) pathways (Tukey-corrected p ≤ 0.05). Alternative splicing events were detected via rMATS, with a focus on isoforms with novel or reference-aligned junctions.

### Gram staining of *Lactobacillus reuteri* GZ-1

Fresh *L. reuteri* GZ-1 colonies grown anaerobically on MRS agar (37 °C, 48 h) were smeared onto glass slides, air-dried, and heat-fixed. Smears were stained with crystal violet (60 s), rinsed, treated with Gram’s iodine (60 s), then decolorized with 95% ethanol (30 s). After rinsing, counterstaining with safranin (30 s) was performed. Slides were examined under oil immersion (100×), revealing characteristic purple Gram-positive rods. Control strains (*E. coli* ATCC 25922) validated staining accuracy.

### Assessment of tolerance to acid and bile salts

The pH of the MRS medium was adjusted to 3.0 using hydrochloric acid. The strains were inoculated into acidified media and cultured in an anaerobic chamber, with a negative control. After 12 h of incubation, the bacterial counts were determined, and the survival ratio relative to the negative control was calculated.

MRS medium was supplemented with bile salts to a final concentration of 0.3% (w/v). Strains were inoculated into the medium and cultured anaerobically, alongside a negative control. The bacterial counts were measured after 12 h, and the growth ratio relative to the negative control was computed.

### Testing for drug resistance

After 12 h of culture in MRS medium, the bacterial strains were diluted 100-fold. A 100 μL aliquot of the diluted suspension was spread onto MRS agar plates, and the antimicrobial susceptibility disks were placed onto the plates. Following 12 h of incubation, the inhibition zone diameters were measured and recorded.

### Phylogenetic analysis of 16S rRNA gene sequences

16S rRNA gene sequences of multiple bacterial strains were retrieved from NCBI. These sequences, along with that of *Lactobacillus reuteri* GZ-1, were aligned using MEGA version 12.0 (https://www.megasoftware.net). A phylogenetic tree was subsequently constructed and visually refined through ChiPlot (https://www.chiplot.online).

### Statistical analysis

Data analysis was performed with GraphPad Prism 8.3.0, and the data are presented as the means ± SDs (one-way ANOVA). Time–to-event data were analysed via the Kaplan–Meier (K–M)/log-rank method. Spearman’s rank correlation coefficient was calculated for correlation analysis. Significance: *p < 0.05; **p < 0.01; ***p < 0.001; ****p < 0.0001.

## Results

### PEDV infection triggers gut microbiota dysregulation in piglets

To explore the relationship between intestinal microbial imbalance and PEDV in Landrace pigs, jejunal contents from PEDV-infected diarrheic piglets (DP, *n* = 10) and healthy controls (HP, *n* = 9) were subjected to 16S rRNA gene amplicon sequencing. Comparative analysis revealed a significantly greater number of operational taxonomic units (OTUs) in DPs (564 OTUs) than in HPs (362 OTUs) (Supplementary Figure S1a). Alpha diversity indices (Chao1, Shannon, Ace, and Simpson) and PCoA of the Bray–Curtis distances revealed distinct microbial community structures between the groups (Supplementary Figures S1b–f).

**Figure 1:**
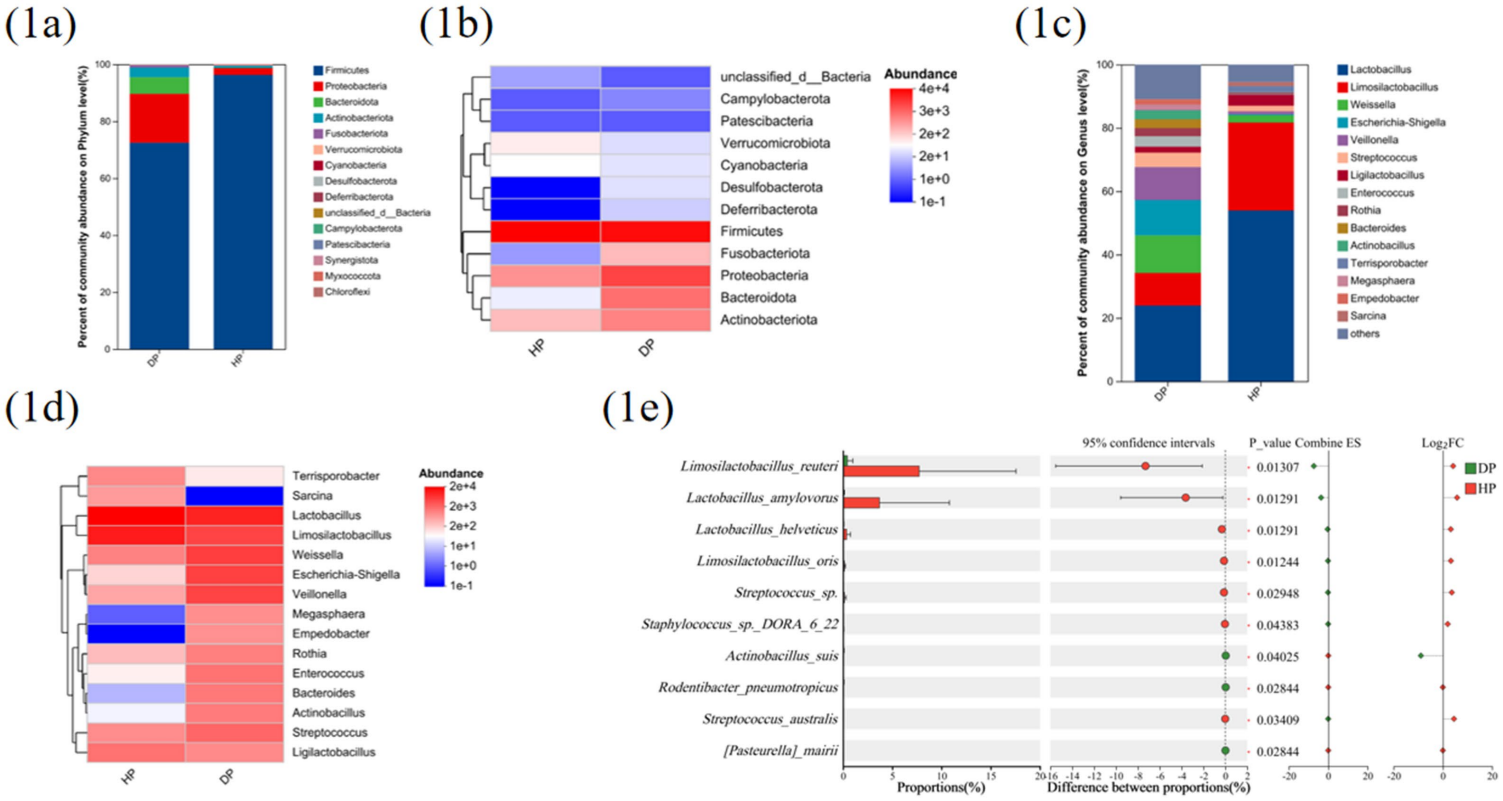
Differences in the gut microbes of PEDV-infected diarrhea piglets (DP) and healthy control piglets (HP). 16S amplicon sequencing results showed differences in phylum **(a,b)** and genus **(c,d)** taxonomic levels between the different groups. **(e)** Differences between groups at the species taxonomic level in metagenomic sequencing results. The results are presented as the means ± SDs, and statistical significance was calculated by one-way ANOVA **(e)**.

Taxonomic profiling at the phylum and genus levels highlighted marked microbial shifts post PEDV infection: the relative abundance of Firmicutes was significantly reduced, whereas the relative abundances of *Bacteroidetes* and *Proteobacteria* were elevated (Figures 1a,b). At the genus level, *Lactobacillus* populations were notably depleted, whereas opportunistic pathogens such as *Escherichia–Shigella*, *Veillonella*, *Empedobacter*, and *Actinobacillus* exhibited marked proliferation (Figures 1c,d).

To resolve species-level dynamics, metagenomic sequencing of small intestinal contents was performed on healthy (*n* = 6) and PEDV-infected (*n* = 6) piglets. Strikingly, commensal species such as *Lactobacillus reuteri*, *Lactobacillus amylovorus*, and *Lactobacillus helveticus,* were markedly depleted in infected piglets (Figure 1e), implying an association between their loss and PEDV pathogenesis.

### Live *Lactobacillus reuteri* transplantation attenuates PEDV-induced enteropathy through viral load reduction and epithelial barrier preservation

**Figure 2:**
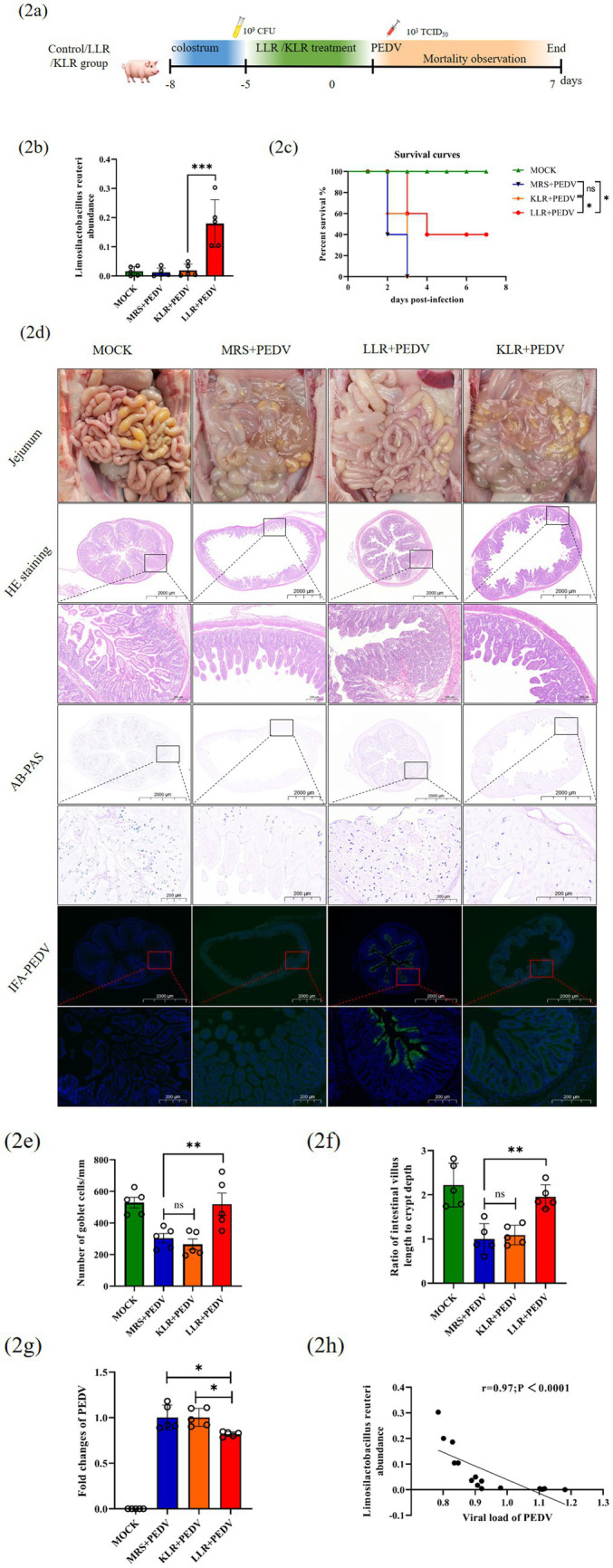
Transplantation of *Lactobacillus reuteri* in piglets against PEDV-induced diarrhea. **(a)** Schematic representation of treatment with MRS, KLR or LLR. **(b)** Abundance statistics of *Lactobacillus reuteri* in different groups by 16S RNA sequencing. **(c)** Survival kinetics analysis after oral administration of MRS, KLR, or LLR followed by PEDV infection (MRS *n* = 5, KLR *n* = 5, LLR *n* = 5).**(d)**Histopathological and pathological changes (HE, AB-PAS and Immunofluorescence staining) in the jejunum of piglets after infection with PEDV were compared at 400 × magnification. Boxed areas are magnified immediately below. Statistical analysis of **(e)** goblet cell counts, **(f)** the villus height-to-crypt depth ratio, and **(g)** PEDV load in the jejunum. Correlation analysis between PEDV content and *Lactobacillus reuteri* abundance **(h)**. Data represent mean ± SD (one-way ANOVA). Significance: *p < 0.05; **p < 0.01; ***p < 0.0001 **(b,e–g)**. Time-to-event data used Kaplan–Meier/log-rank methods(c). Spearman’s rank correlation coefficient was carried out for correlation analysis **(h)**.

To evaluate the antiviral potential of jejunal-derived commensals, *Lactobacillus helveticus* (*n* = 2), *Lactobacillus reuteri* (*n* = 5), and *Lactobacillus amylovorus* (*n* = 2) were isolated from healthy piglet jejuna via De Man, Rogosa, and Sharpe (MRS) media under anaerobic conditions. The candidate strains were subjected to functional screening: acid/bile salt tolerance assays were used to assess colonization potential, whereas antibiotic resistance profiling was used to evaluate transmission risk (Supplementary Figures S2a–c). *Lactobacillus reuteri* GZ-1 (*L. reuteri*), identified as the most promising candidate, was selected to establish transplantation models alongside MRS medium controls and killed *L. reuteri* (KLR) via pasteurization (Figure 2a).

16S rRNA gene amplicon sequencing confirmed successful jejunal colonization in live *L. reuteri* (LLR)-treated piglets, with *L. reuteri* RNA levels significantly exceeding those in MRS controls. KLR failed to colonize, as evidenced by the unchanged LR relative abundance (Figure 2b). Principal coordinate and dendrogram cluster analyses revealed that LLR/KLR transplantation did not alter the overall microbiota composition, indicating *L. reuteri*-specific activity rather than community-level modulation (Supplementary Figures S2d–g).

Following PEDV challenge, LLR but not KLR or MRS significantly alleviated diarrhea and mortality (*p* < 0.01), with all KLR/MRS piglets succumbing by day 3 post infection (Figure 2c). The surviving LLR piglets (euthanized at 7 dpi) presented preserved jejunal architecture via H&E staining and maintained goblet cell density via AB-PAS staining, in contrast with severe epithelial damage in the controls (Figures 2d–f).

Consistent with its barrier-protective effects, LLR treatment reduced jejunal PEDV RNA loads compared with those of KLR (Figure 2g). The viral load was inversely correlated with the relative abundance of LRs (Figure 2h), indicating that *L. reuteri*-driven viral suppression is a key mechanism underlying intestinal protection.

### *L. reuteri* exerts anti-PEDV effects through indirect rather than direct viral interactions

**Figure 3:**
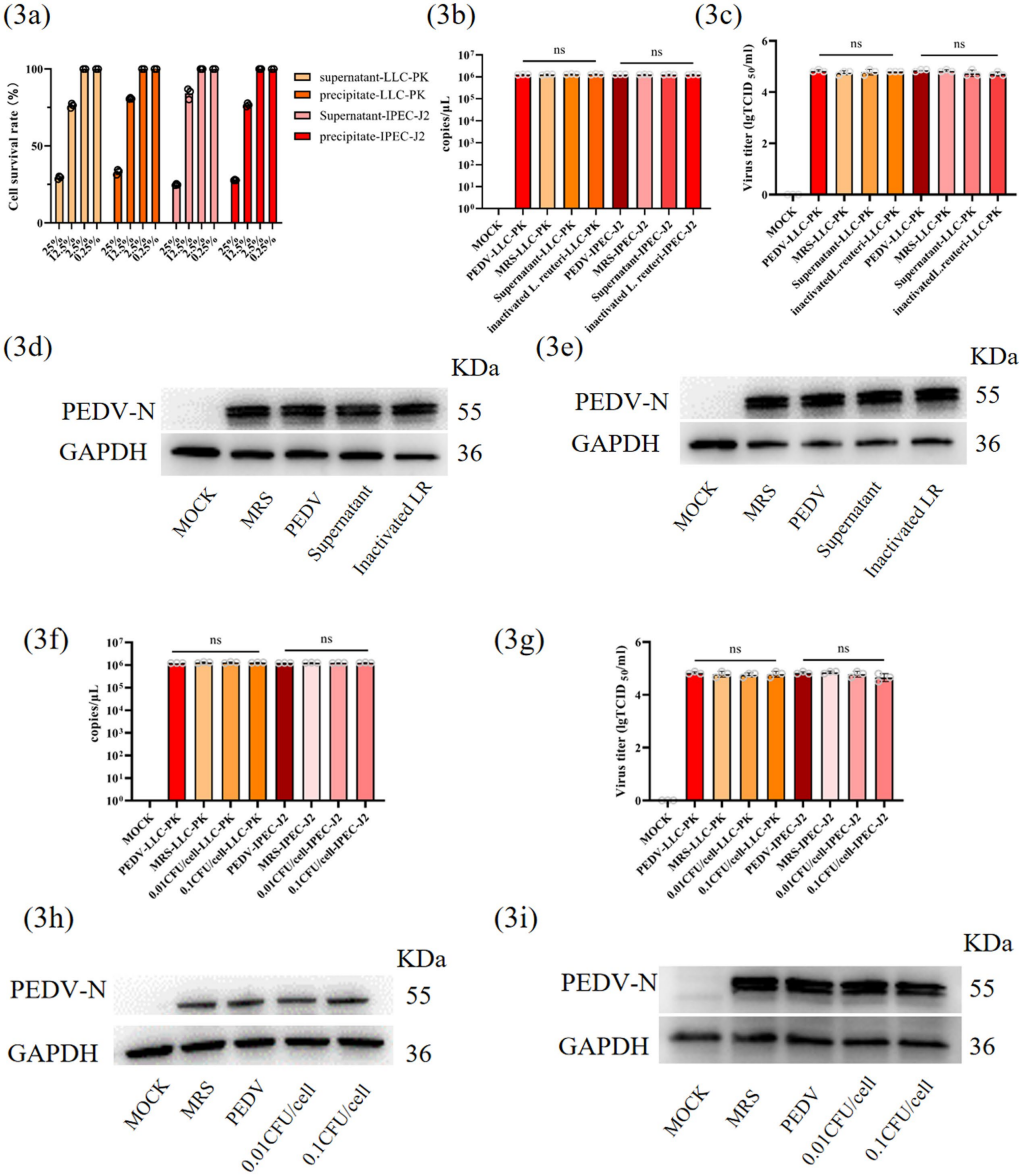
Inhibitory effects of *Lactobacillus reuteri* GZ-1 on PEDV replication in LLC-PK1 and IPEC-J2 Cells. **(a)** Safe concentrations of different components of *Lactobacillus reuteri* solution in vitro. Statistics of absolute mRNAlevels **(b)** and PEDV titres **(c)** after 24 hpi by pretreatment with different components of *Lactobacillus reuteri*. The expression levels of PEDV-N in LLC-PK1 **(d)** and IPEC-J2 **(e)** were determined. Statistics of absolute mRNA levels **(f)** and PEDV titres **(g)** after 24hpi by pretreatment with different CFU of *Lactobacillus reuteri*. The expression levels of PEDV-N in LLC-PK1 **(h)** and IPEC-J2 **(i)** cells were determined. Data represent mean ± SD (one-way ANOVA). Significance: *p < 0.05; **p < 0.01; ***p < 0.0001 **(b,c,f,g)**.

To further investigate the mechanism underlying *L. reuteri*-mediated inhibition of PEDV replication, pasteurized *L. reuteri* preparations were cocultured with PEDV in two distinct epithelial cell models (IPEC-J2 and LLC-PK1 cells). As previously described (Xie et al., 2023), both the supernatant and the cellular precipitate derived from inactivated bacterial cultures were separated and incubated with PEDV at physiologically safe concentrations (Figure 3a). Subsequent quantification of the viral load through nucleic acid detection, viral protein analysis, and TCID_50_ assays 24 h post infection (hpi) revealed no significant antiviral effects from either inactivated bacterial fraction (Figures 3b–e).

This unexpected outcome prompted subsequent evaluation of the antiviral potential of live *L. reuteri*. Bacterial suspensions standardized to 1 × 10^9^ CFU/mL were administered at two cellular ratios (0.01 and 0.1 CFU/cell) during 2-h pretreatment protocols prior to PEDV challenge (0.1 MOI). Despite rigorous quantification through parallel virological assessments (nucleic acid quantification, viral protein detection, and TCID_50_ determination), neither bacterial concentration suppressed viral replication (Figures 3f–i).

These collective findings suggest that the anti-PEDV activity of *L. reuteri* likely operates through host-mediated pathways or indirect modulatory effects rather than through direct virucidal action or immediate interference with viral replication.

### *L. reuteri* promotes the biosynthesis of HDCA

**Figure 4:**
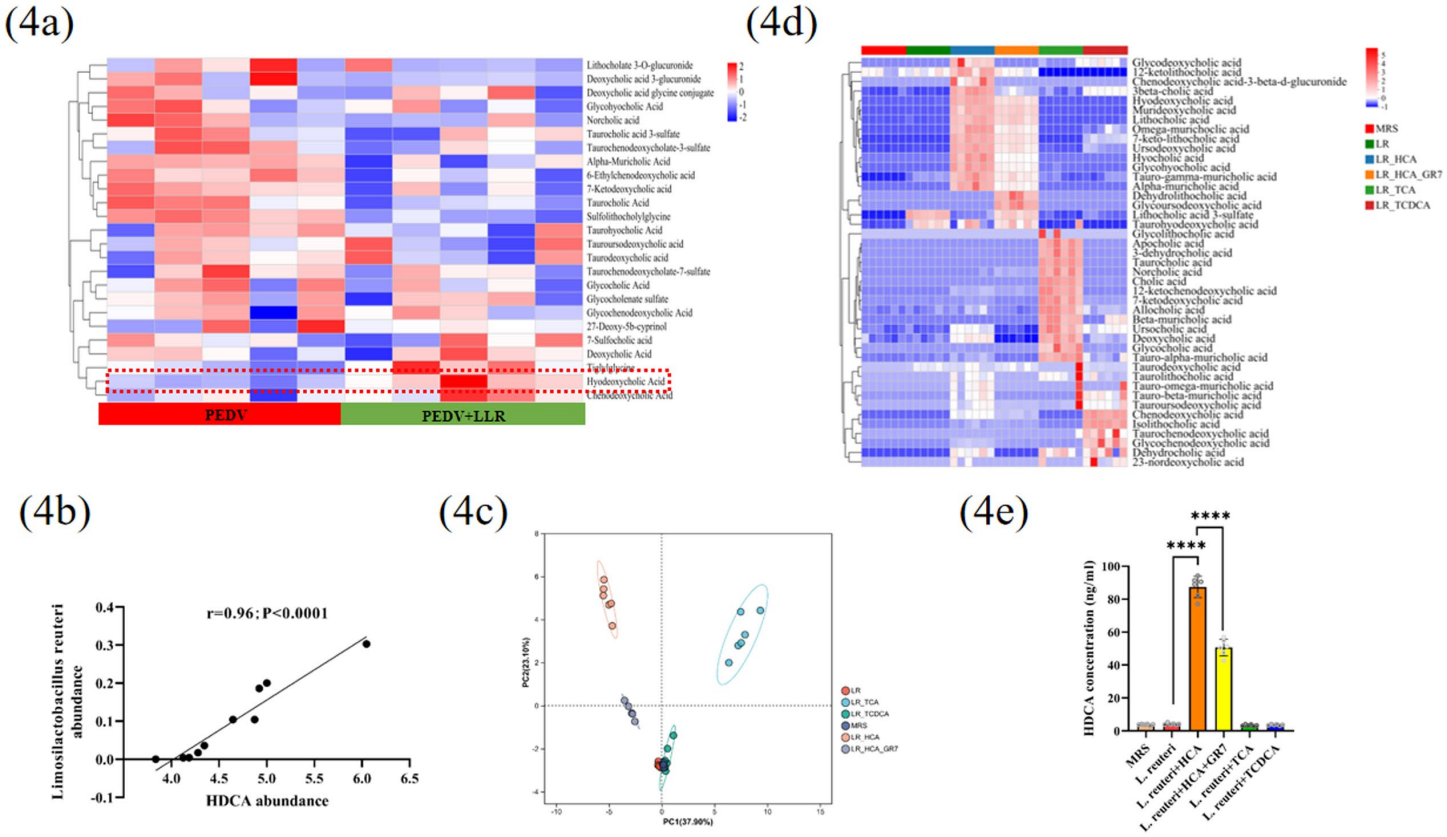
Interaction analysis of *Lactobacillus reuteri* and HDCA across experimental models. **(a)** Cluster heatmap analysis of bile acid aggregation from untargeted metabolomics results in PEDV-infected piglets with or without *Lactobacillus reuteri* transplanted. **(b)** Correlation analysis of *Lactobacillus reuteri* colonization density and HDCA accumulation in jejunal contents. Principal component **(c)** and Cluster heatmap analysis **(d)** were performed on targeted bile acid profiling data obtained from *Lactobacillus reuteri* coculture fermentation supplemented with TCA, TCDCA, HCA or HCA + GR7. **(e)** Statistical analysis was performed to quantify HDCA content in fermentation broth of different treatment groups. Data represent mean ± SD (one-way ANOVA). Significance: *p < 0.05; **p < 0.01; ***p < 0.0001 **(e)**. Spearman’s rank correlation coefficient was carried out for correlation analysis **(b)**.

Microbes regulate host metabolic and immune functions through their metabolites. To determine whether *L. reuteri* influences PEDV infection via metabolites, we performed untargeted metabolomics analysis of jejunal contents from PEDV-infected piglets with or without *L. reuteri* transplantation. Partial least squares discriminant analysis (PLS-DA) revealed that PEDV infection significantly altered the jejunal metabolic profile (Supplementary Figure S4a). Differential analysis revealed 3,076 common metabolites, with 759 showing significant changes (110 upregulated, 649 downregulated; Supplementary Figures S4b,c). Metabolomic profiling highlighted a cluster of differentially abundant bile acids (BAs), including taurocholic acid (TCA), HDCA, and chenodeoxycholic acid (CDCA), with HDCA being the most significantly altered (Figure 4a). Quantitative analysis revealed a strong positive correlation between *L. reuteri* colonization density and HDCA accumulation (Spearman’s *r* = 0.96, *p* < 0.0001), suggesting microbiota-driven HDCA biosynthesis in the gut (Figure 4b).

HDCA, a microbially modified secondary bile acid, is derived from the primary bile acid hyocholic acid (HCA) via gut microbial metabolism (Eyssen et al., 1999). To investigate the relationship between *L. reuteri* and HDCA, we conducted targeted metabolomics on *L. reuteri* fermentation broth with or without HCA supplementation (MRS medium as a control). Principal component analysis (PCA) confirmed the experimental reproducibility, revealing clear clustering within treatment groups and separation between cohorts (Figure 4c). Sample cluster heatmap analysis further supported these findings (Figure 4d). Notably, HDCA production was significantly increased when *L. reuteri* was cocultured with 100 μM HCA (Figure 4e), confirming its enzymatic capacity to convert HCA to HDCA.

Bile salt hydrolases (BSHs) and 7α-dehydroxylase are key enzymes in primary-to-secondary bile acid conversion (Li et al., 2024). To characterize *L. reuteri* bile acid metabolism, we performed targeted UHPLC–MS/MS metabolomics on fermentation broth supplemented with 100 μM TCA or TCDCA, which produces CA and CDCA, respectively (Figures 4c,d and Supplementary Figures S4d,e). These results confirmed *L. reuteri*’s BSH activity, and the BSH gene was further validated via PCR (Supplementary Figure S4f). To assess the role of BSH in HDCA production, we cocultured HCA with the BSH inhibitor gut-restricted-7 (GR7), which significantly reduced—but did not abolish—HDCA production (Figures 4c–e). This partial inhibition suggests complementary mechanisms, potentially involving 7α-dehydroxylase-mediated.

### HDCA inhibited PEDV replication in LLC-PK1 and IPEC-J2 cells

**Figure 5:**
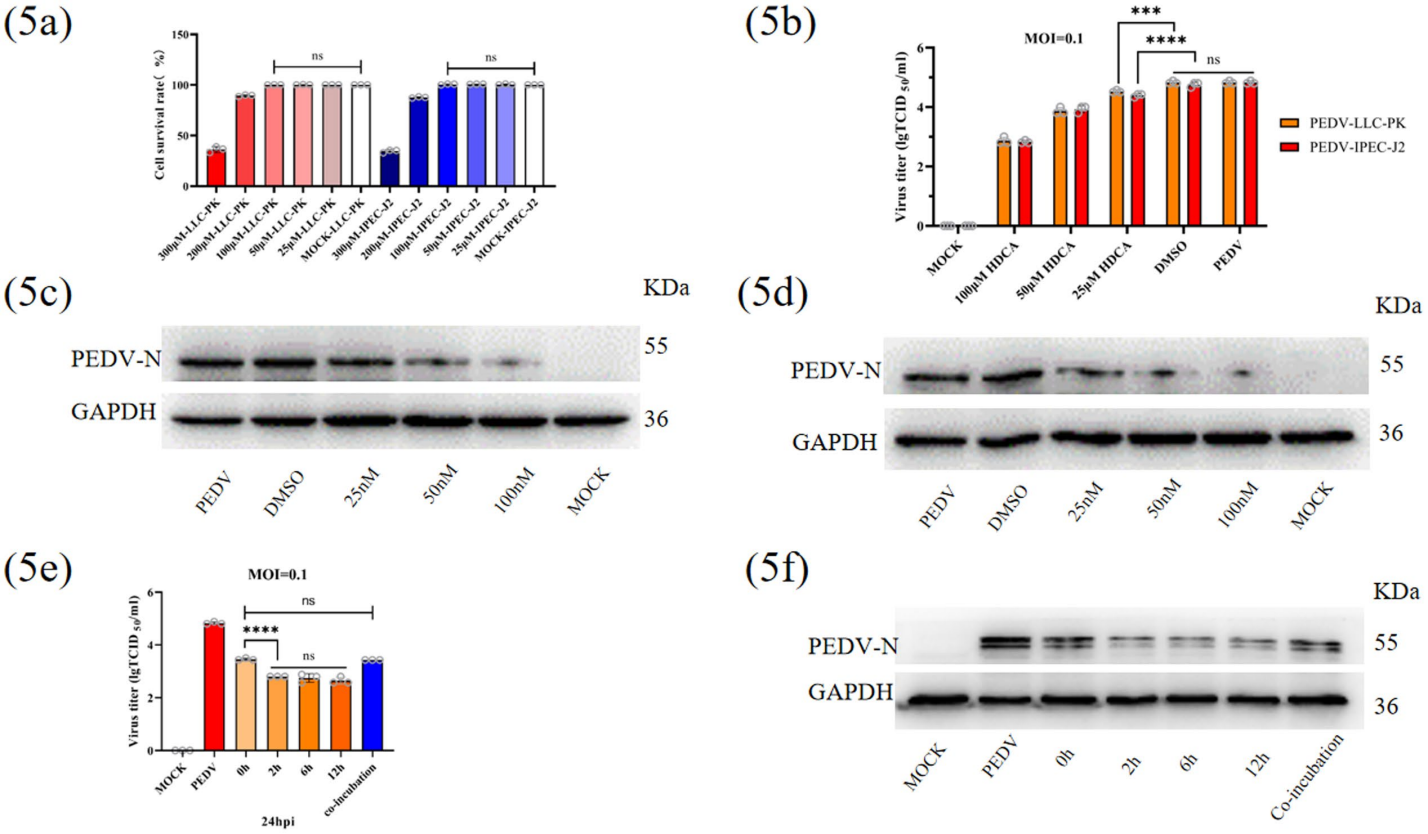
Inhibitory effects of HDCA on PEDV replication in LLC-PK1 and IPEC-J2 Cells. **(a)** Safe concentration of HDCA in vitro. **(b)** Statistics of PEDV titre with different concentrations of HDCA. The expression levels of PEDV-N in LLC-PK1 **(c)** and IPEC-J2 **(d)** were determined. PEDV titre **(e)** and PEDV-N expression **(f)** assay with different treatment time or method of HDCA in IPEC-J2. Data represent mean ± SD (one-way ANOVA). Significance: *p < 0.05; **p < 0.01; ***p < 0.0001 **(a,b,e)**.

Some secondary bile acids have been reported to suppress viral replication in previous studies. For example, CDCA and DCA inhibit murine norovirus-1 infection (Grau et al., 2020), whereas the bile salt derivative 6ECDCA significantly reduces hepatitis B virus replication (Radreau et al., 2016). To determine whether HDCA protects against PEDV by inhibiting viral replication, we first assessed its cytotoxicity in LLC-PK1 and IPEC-J2 cells and found a TC_50_ value of 100 μM (Figure 5a). The cells were then pretreated with varying concentrations of HDCA (within safe limits) for 2 h before PEDV infection (0.1 MOI). After 24 h, viral titres and N protein expression levels were measured. The results demonstrated that HDCA suppressed PEDV replication in a dose-dependent manner, with maximal inhibition observed at 100 μM HDCA (Figures 5b–d).

To further evaluate the inhibition pattern of HDCA, IPEC-J2 cells were pretreated with 100 μM HDCA for different durations prior to PEDV infection. At 24 hpi, experimental analyses demonstrated that HDCA pretreatment significantly suppressed viral replication, indicating that HDCA-mediated antiviral activity originates from host cell immunomodulation rather than direct virucidal effects (Figures 5e–f). To confirm this hypothesis, PEDV was preincubated with HDCA at 37 °C for 2 h prior to cell inoculation. The antiviral activity was similar to that in the nonpretreated group, further suggesting that the antiviral mechanism of HDCA relies on cellular pathways rather than direct virucidal effects (Figures 5e–f).

### HDCA and *Lactobacillus reuteri* protect piglets against PEDV infection via jejunal HDCA accumulation

**Figure 6:**
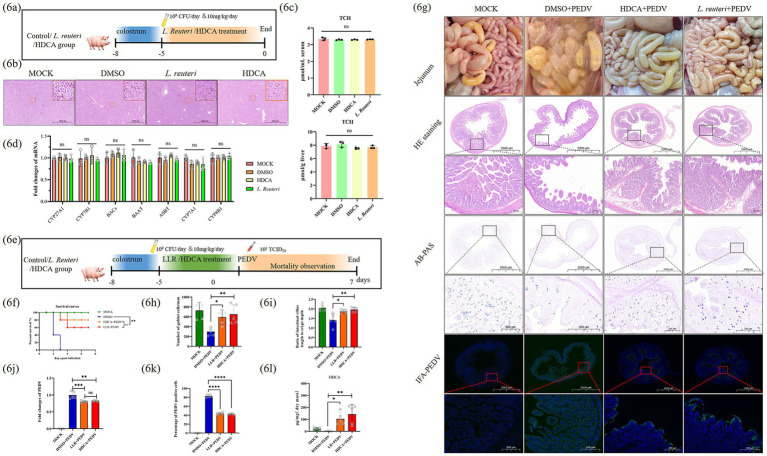
*Lactobacillus reuteri* and HDCA attenuate PEDV-induced diarrhea in piglets. **(a)** Schematic representation of safety assessment with DMSO, HDCA or *Lactobacillus reuteri*. **(b)** H&E staining of the liver at 400 × magnification. Boxed areas are immediately magnified in the upper right. **(c)** Cholesterol concentrations in liver and serum. **(d)** Transcript level analysis of key bile acid metabolism regulators. **(e)** Schematic representation of treatment with DMSO, *Lactobacillus reuteri* or HDCA. **(f)** Survival kinetics analysis after oral administration of DMSO, *Lactobacillus reuteri* or HDCA followed by PEDV infection. **(g)** Histopathological and pathological changes (H&E, AB-PAS, and Immunofluorescence staining) in the jejunum of piglets after infection with PEDV were compared at 400 × magnification. Boxed areas are magnified immediately below. Statistics of goblet cell counts **(h)** and the ratio of villus length to crypt depth **(I)** in jejunum. **(j)** The relative RNA of PEDV in jejunum. **(k)** The proportion of PEDV-positive cells in the intestinal villi of the jejunum. **(l)** HDCA content statistics of jejunal contents. Data represent mean ± SD (one-way ANOVA). Significance: *p < 0.05; **p < 0.01; ***p < 0.0001 **(c,d,h–i)**. Time-to-event data used Kaplan–Meier/log-rank methods **(f)**.

During hepatic cholesterol metabolism, primary bile acids are synthesized and transported to the intestinal lumen through the enterohepatic circulation, where the gut microbiota transforms them into secondary bile acids. Growing evidence suggests that secondary bile acids combat viral pathogenesis through multiple mechanisms—for example, LCA has protective effects in animal models (Xing et al., 2024), whereas TLCA inhibits SFTSV replication and reduces inflammation *in vitro* (Zheng et al., 2024). To evaluate the antiviral potential of HDCA against PEDV *in vivo*, we established a neonatal piglet model. Piglets received oral DMSO (control), *L. reuteri*, or HDCA for 5 days to assess safety (Figure 6a). Intestinal tissue examination and liver histopathology (H&E staining) confirmed that there was no treatment-related hepatotoxicity (Figure 6b). Since bile acids are derived from hepatic cholesterol catabolism (Chiang, 1998; Chiang, 2004), we measured cholesterol levels in the liver and serum and found no significant differences between the groups (Figure 6c). Additionally, the transcript levels of bile acid metabolism regulators (CYP27A1, BAAT, CYP7B1, BACs, ASBT, CYP7A1, and CYP8B1) remained stable in hepatic or ileal tissues, indicating that HDCA administration does not disrupt cholesterol homeostasis or bile acid metabolism (Figure 6d).

After 5 days of pretreatment, piglets were orally challenged with PEDV (1,000 TCID₅₀), and mortality was monitored for 7 days (Figure 6e). Both HDCA and *L. reuteri* significantly reduced PEDV-induced mortality (Figure 6f). Gross pathology revealed diminished diarrhea in HDCA-treated piglets, mirroring the protective effects of *L. reuteri.* H&E and AB-PAS staining revealed preserved intestinal mucosal integrity, including intact goblet cells and villus structure, in the treated groups (Figures 6g–i). Immunofluorescence staining and viral load analysis confirmed that PEDV replication was suppressed in the HDCA- and *L. reuteri*-treated jejunum (Figures 6g,j,k).

LC–MS/MS-based bile acid metabolomics of intestinal contents revealed substantial HDCA accumulation in both treatment groups (HDCA: 105-fold increase; *L. reuteri*: 76.9-fold vs. DMSO controls) (Figure 6l), despite unaltered systemic bile acid profiles. These findings demonstrate that the antiviral effects of HDCA stem from local jejunal enrichment rather than systemic metabolic changes.

### HDCA inhibits PEDV replication via the TGR5-cAMP signalling axis

**Figure 7:**
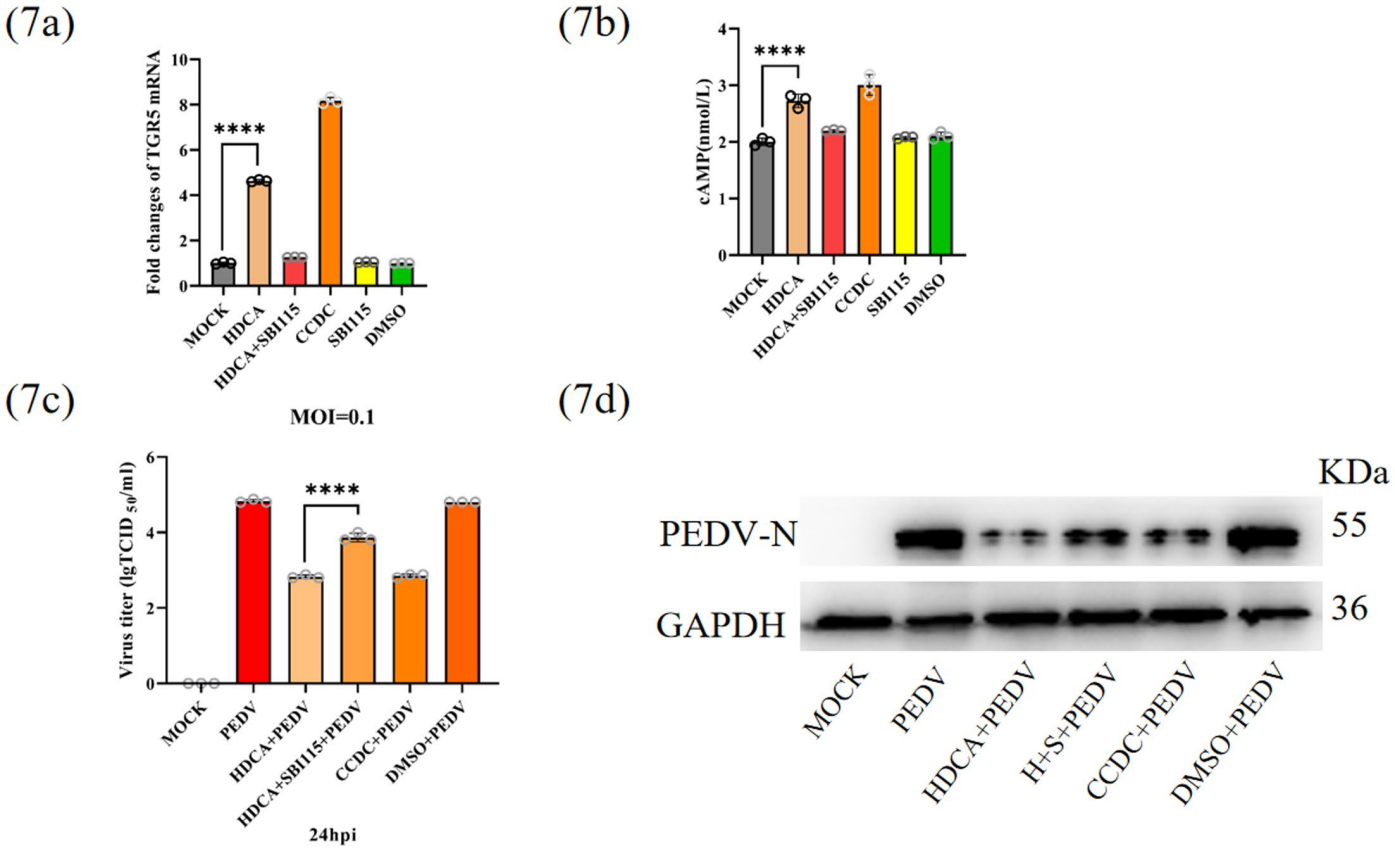
HDCA inhibits PEDV replication via TGR5-cAMP signalling axis. Intracellular TGR5 mRNA **(a)** and cAMP **(b)** content were quantified 2 h posttreatment with various experimental conditions. Cells were infected with PEDV for 24 h after pretreatment, and the expression of PEDV RNA **(c)** and N-protein **(d)** was detected (H + S + PEDV represents hdca+sbi115 + pedv). Data represent mean ± SD (one-way ANOVA). Significance: *p < 0.05; **p < 0.01; ***p < 0.0001 **(a–c)**.

HDCA, a secondary bile acid and potent agonist of GPBAR1/TGR5 (Sato et al., 2008), was used to delineate the molecular mechanism underlying TGR5 activation. Using the TGR5-specific agonist CCDC and antagonist SBI-115 as pharmacological controls, we examined both TGR5 mRNA expression by qRT–PCR and intracellular cAMP levels by ELISA in IPEC-J2 cells. HDCA treatment significantly increased TGR5 mRNA expression (2.5-fold) and cAMP levels (3.1-fold), demonstrating agonist activity comparable to that of CCDC (Figures 7a,b). Viral challenge experiments functionally validated these findings: TGR5 activation with CCDC mimicked the antiviral effects of HDCA, whereas pharmacological inhibition with SBI-115 attenuated HDCA-mediated protection. These results were confirmed through measurements of PEDV RNA and N protein expression levels (Figures 7c,d).

### Multiomics analysis reveals that HDCA modulates the NF-κB and ISG15 pathways against PEDV

**Figure 8:**
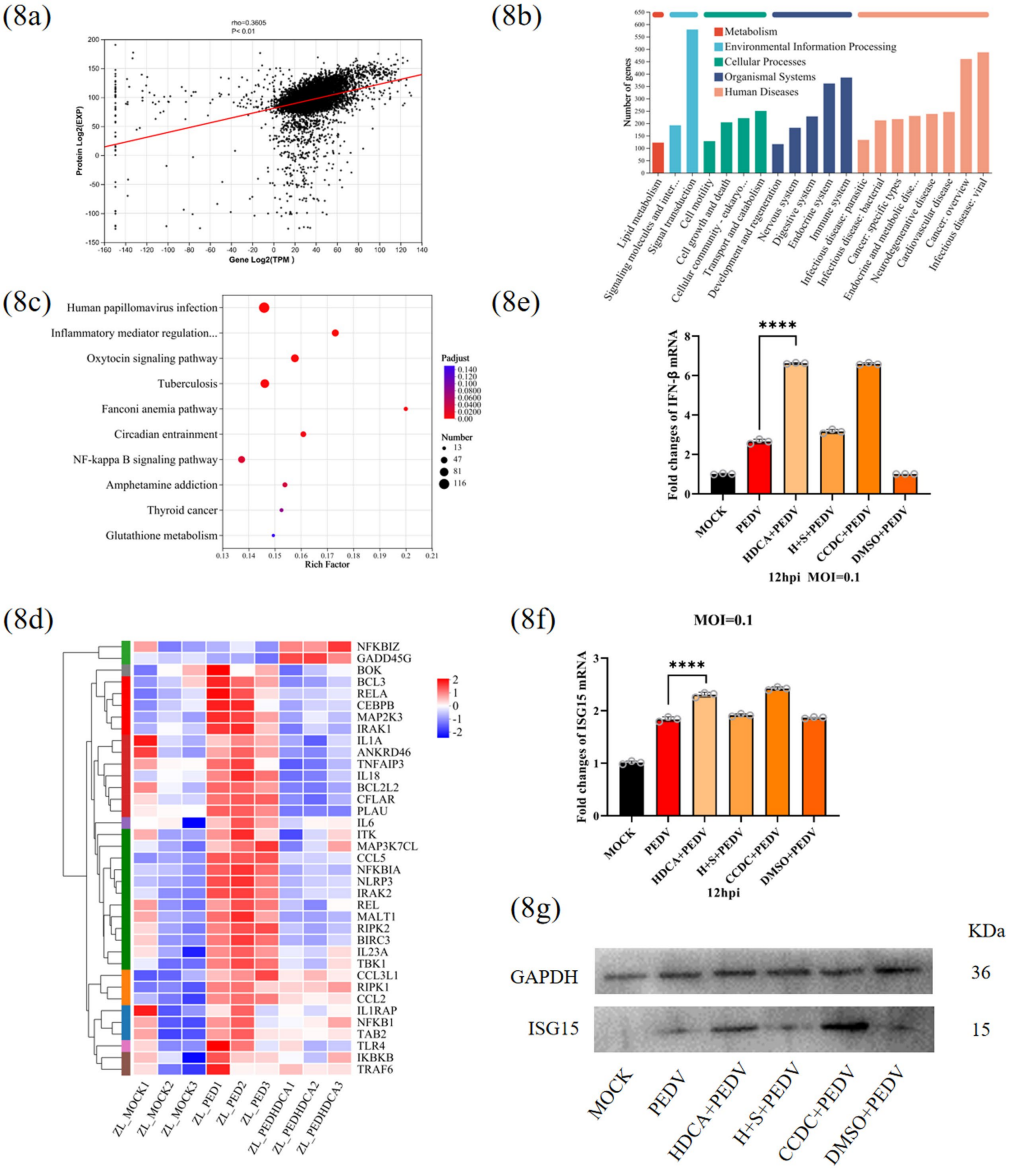
Multiomics analysis reveals HDCA modulates NF-κB and ISG15 pathways against PEDV. **(a)** The statistical analysis of the protein-transcript association dataset. **(b)** KEGG functional annotation analysis on transcriptomic datasets. **(c)** KEGG pathway enrichment analysis of the transcriptomic datasets. **(d)** Cluster analysis of NF-κB pathway related genes. **(e)** Relative mRNA levels of IFN-β in IPEC-J2 cells with various experimental conditions. ISG15 mRNA **(f)** and protein **(g)** expression level in IPEC-J2 cells with various experimental conditions (H + S + PEDV represents HDCA+SBI115 + PEDV).

To characterize HDCA’s antiviral mechanisms comprehensively, we employed a multiomics approach combining RNA sequencing (RNA-seq) and liquid chromatography–tandem mass spectrometry (LC–MS/MS) of PEDV-infected IPEC-J2 cells with or without HDCA pretreatment. Protein–transcript association analysis revealed a moderate but significant positive correlation (Pearson’s *ρ* = 0.3506, *p* < 0.01) between the proteomic and transcriptomic profiles (Figure 8a). PCA demonstrated strong experimental reproducibility, showing tight intragroup clustering and clear intergroup separation (Supplementary Figures S8a,b). Cross-sample analysis revealed 13,203 genes and 6,902 proteins, with 92.3% of the genes (12,189) and 99.3% of the proteins (6,854) shared across groups (Supplementary Figures S8c,d). Differential expression analysis (transcripts: |log2FC| ≥ 0.585 and *p* < 0.05; proteins: |log2FC| ≥ 0.263 and *p* < 0.05) revealed 2,980 DEGs (1,782 upregulated, 1,198 downregulated) and 607 DEPs (449 upregulated, 158 downregulated) (Supplementary Figures S8e,f). KEGG annotation highlighted signal transduction as the most significantly enriched subcategory, containing 189 DEGs and 68 DEPs (Figure 8b; Supplementary Figure S8g). Pathway analysis revealed 212 significantly enriched pathways (*p* < 0.05), with NF-κB signalling being particularly prominent. Key NF-κB regulators (RELA and NFKB1) were significantly downregulated in HDCA-treated cells (Figures 8c,d). The “infectious disease: viral” pathway involving ISG15 was also enriched at both omics levels, with the ISG15 protein significantly upregulated (Figure 8c; Supplementary Figures S8h–j). Preliminary validation revealed increased IFN-*β* and ISG15 expression in HDCA-treated cells, suggesting their involvement in HDCA antiviral action (Figures 8e–g).

## Discussion

Our study demonstrated that *L. reuteri* protects piglets from PEDV-induced mortality through HDCA-mediated mechanisms. Mechanistic investigations revealed the dual protective effects of HDCA: suppressing NF-κB signalling to mitigate inflammation while increasing ISG15 expression to inhibit viral replication. Given the jejunal tropism of PEDV (Li et al., 2016), dual-omics analysis (16S rRNA gene amplicon and metagenomic sequencing) of jejunal digesta revealed an 18-fold depletion of *L. reuteri* in fatal PEDV cases compared with healthy controls (p < 0.05), establishing its keystone species status (Figure 1e). While some studies have reported increased lactobacilli during PEDV infection (Xing et al., 2024; Huang et al., 2019; Huang et al., 2018), our findings align with others showing depletion (Li et al., 2022; Wang et al., 2024). We speculate that the discrepancies across studies may arise from methodological variations in sampling protocols (mucosal vs. luminal) or infection stages.

The limitations of this study include the inability to use antibiotics precolonized with *L. reuteri* due to its antibiotic sensitivity and neonatal susceptibility to PEDV (Liu et al., 2016; Sun et al., 2012), preventing monocolonization validation. However, our model revealed selective *L. reuteri* enrichment without disruption of the overall microbiota (Supplementary Figures S2d–g), indicating that monotherapy with the strain was sufficient to alleviate PEDV-induced enteropathy. This finding is analogous to the reported inhibition of PEDV infection by *Lactobacillus rhamnosus* GG (Wang et al., 2024).

Previous reports suggest that bacteria or their metabolites inhibit PEDV (Huang et al., 2021; Chang-Liao et al., 2020). However, neither heat-inactivated *L. reuteri* nor heat-inactivated *L. reuteri* or *L. reuteri* supernatant showed antiviral activity (Figures 3b–e), nor did viable cells (Figures 3f–i) in our study. It is well established that microbiota-generated metabolites persistently engage with their cognate receptors to modulate immune function (Yang and Cong, 2021). Therefore, we conducted untargeted metabolomics, which revealed significantly elevated bile acids, particularly HDCA, in *L. reuteri*-treated piglets (Figure 4a), with a strong correlation between *L. reuteri* relative abundance and HDCA levels (Spearman’s *r* = 0.96, *p* < 0.0001) (Figure 4b). Through *in vitro* coculture experiments, we demonstrated that *L. reuteri* actively converts HCA to HDCA (Figures 4d,e), which was confirmed *in vivo* (Figures 4b, 6l). It has been suggested that BSHs and 7α-dehydroxylase are recognized as key enzymatic mediators of primary-to-secondary bile acid transformation (Li et al., 2024). Although BSHs were identified (Supplementary Figures S4d–f), incomplete HDCA suppression by BSH inhibitors suggests 7α-dehydroxylase involvement, although genomic annotation and homology-based functional prediction of *L. reuteri* revealed no identifiable 7α-dehydroxylase. While previous reports by Wang et al. revealed correlations between *L. reuteri* and other bile acids (DCA, LCA) in porcine feces (Xing et al., 2024), our study is the first to establish a novel association between jejunal *L. reuteri* colonization and HDCA accumulation.

HDCA, a major porcine BA with therapeutic potential for metabolic and inflammatory disorders (Li et al., 2016; Sun et al., 2012; Kuang et al., 2023; Spinelli et al., 2016), has been shown to have immunomodulatory effects on colitis (Watanabe et al., 2022), growth performance (Zong et al., 2019) and intestinal inflammation (Tao et al., 2025). While previous studies focused on host interactions, we first demonstrated HDCA’s antiviral activity against PEDV in vitro (Figure 5b–d) and in vivo (Figure 6g,j,k), which was supported by reports of HDCA-containing traditional medicines inhibiting SARS-CoV-241 (Ma et al., 2022).

To elucidate the underlying mechanism by which HDCA inhibits PEDV replication, we focused on TGR5, which was first identified in 2002 as an HDCA sensor (Maruyama et al., 2002; Zheng et al., 2021; Si et al., 2024). Mechanistic studies revealed that HDCA activates TGR5, increasing cAMP levels (Figures 7a,b). TGR5 inhibition attenuated the antiviral effect of HDCA, whereas TGR5 agonism mimicked it (Figures 7c,d). These findings compellingly establish TGR5 as the pivotal receptor mediating the anti-PEDV activity of HDCA. Similar to our study, emerging evidence suggests TGR5 exerts antiviral effects by potentiating IFN-I signalling pathways (Xiong et al., 2018). We further performed integrated proteomic and transcriptomic profiling and revealed that HDCA-mediated protection against PEDV involves the suppression of NF-κB-driven inflammatory responses (Figure 8b-d). In line with these findings, the TGR5-specific agonist INT-777 mediates anti-inflammatory effects via cAMP-mediated NF-κB pathway inhibition (Pols et al., 2011). Zhu et al. (2022) demonstrated that the antineuroinflammatory effect of HDCA involves TGR5-initiated AKT/NF-κB cascade modulation. Xie et al. (2023) reported that GCDCA suppresses NF-κB-mediated inflammatory responses. Our multiomics analysis further revealed that HDCA may suppress PEDV replication by activating the type I interferon (IFN) pathway, thereby upregulating interferon-stimulated gene 15 (ISG15), a potent antiviral effector induced by type I interferons that disrupts viral translation, replication, and egress and has been shown to inhibit PEDV replication (Figures 8g–i) (Schneider et al., 2014; Gao et al., 2024). Experimental validation confirmed the HDCA-mediated induction of IFN-*β* and subsequent ISG15 activation (Figures 8e–g), providing preliminary evidence for the role of ISG15 in curbing PEDV replication. Overall, we propose a dual mechanism: (1) NF-κB suppression reduces inflammation, and (2) the TGR5-cAMP-IFNβ-ISG15 axis enhances antiviral defense. While these findings suggest a novel immune–metabolic mechanism through which microbiota-derived bile acids combat PEDV infections, further functional studies are imperative to fully delineate the molecular interplay within this proposed axis.

## Conclusion

**Figure 9:**
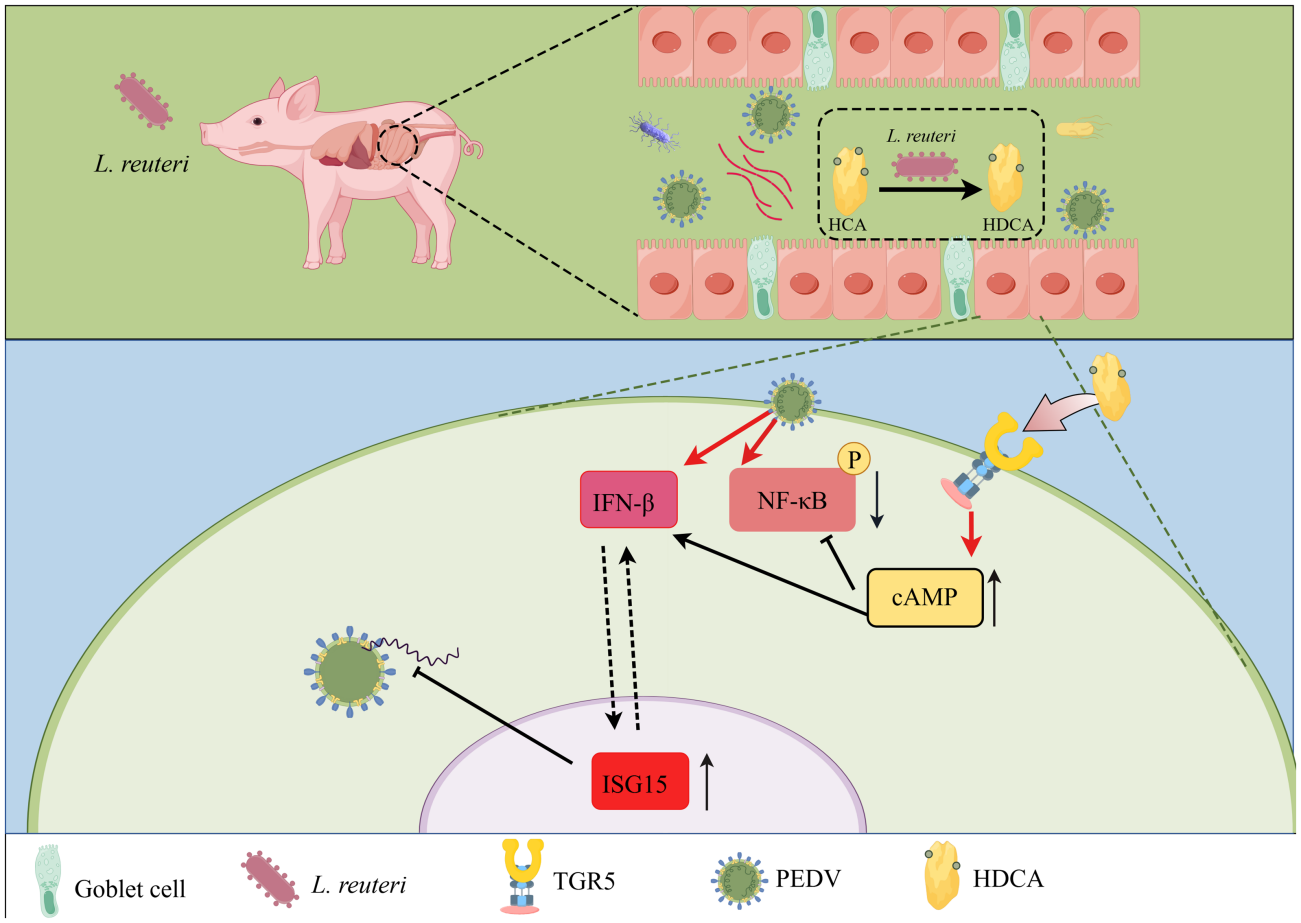
Mechanisms of PEDV infection alleviation by *Lactobacillus reuteri. Lactobacillus reuteri*, when introduced into 3-day-old piglets, colonizes the intestinal tract and enhances the production of HDCA, thereby mitigating PEDV infection. This intervention manifests in increased jejunal goblet cell counts, elevated villus-to-crypt ratios, and reduced PEDV loads in jejunum. Mechanistically, *Lactobacillus reuteri* significantly promotes HDCA metabolism, and in vitro studies confirm that HDCA inhibits PEDV replication in LLC-PK1 and IPEC-J2 cells. Further multiomics analyses predict that HDCA alleviates PEDV infection through dual pathways: (1) attenuating NF-κB-driven inflammatory responses and (2) suppressing PEDV replication via ISG15-mediated antiviral activity.

This study demonstrated that *L. reuteri* colonization protects piglets from lethal PEDV infection, primarily through the microbial metabolite HDCA. We establish a novel link between jejunal *L. reuteri* infection in fatal PEDV cases and its ability to actively convert primary bile acids to HDCA in vivo. Crucially, we identified HDCA as a potent antiviral agent against PEDV that acts via the bile acid receptor TGR5. Mechanistically, HDCA has dual protective effects: (1) it activates the TGR5-cAMP axis, leading to the induction of IFN-β and subsequent upregulation of the potent antiviral effector ISG15, thereby directly inhibiting viral replication; (2) it suppresses NF-κB-mediated inflammatory signalling, mitigating PEDV-induced enteropathy (Figure 9a). This work reveals a previously unrecognized immune-metabolic pathway in which a keystone gut bacterium (*L. reuteri*) generates a bioactive bile acid (HDCA) that combats enteric viral infection through coordinated immunomodulation (NF-κB suppression) and enhanced antiviral defense (TGR5-cAMP-IFNβ-ISG15 axis). These findings highlight microbiota-derived bile acids, particularly HDCA, as critical mediators of host defense against PEDV and potentially other enteric coronaviruses, suggesting novel therapeutic avenues. However, further functional studies are needed to fully elucidate the molecular details of this axis.

## Data Availability

The datasets presented in this study can be found in online repositories: NCBI, BioProject accession PRJNA1265437, PRJNA1268767 and PRJNA1272263; iProX, project ID PXD065241; and MetaboLights, study identifier MTBLS12632, MTBLS12640, and MTBLS12641.

## Supplementary material

SUPPLEMENTARY FIGURE S1 Differences in the gut microbes of PEDV-infected diarrhea piglets (DP) and healthy control piglets (HP). **(a)** Comparative analysis of intestinal microbial OTUs of PEDV-infected diarrhea piglets (DP) and healthy control piglets (HP). Comparative analysis of Chao1 **(b)**, Shannon **(c)**, Ace **(d)**, and Simpson **(e)** indices among different groups. **(f)** PCoA based on weighted bray curtis to identify microbial structural changes.

SUPPLEMENTARY FIGURE S2 Evaluation of *L. reuteri* GZ-1 properties and their impact on jejunal microbiota in piglets following transplantation. The acid resistance **(a)**, bile salt resistance **(b)** and antibiotic resistance **(c)** of the strains were tested. Nonmetric multidimensional **(d)**, principal co-ordinates analysis **(e)**, community barplot analysis **(f)** and hierarchical clustering cluster analysis **(g)** of jejunal contents in each group. Data represent mean ± SD **(a-c)**.

SUPPLEMENTARY FIGURE S4 Interaction analysis of *L. reuteri* and HDCA across experimental models. The Partial Least Squares Discriminant **(a)**, Venn diagram **(b)** and Differential expression **(c)** analysis was performed using untargeted metabolomics sequencing of jejunal contents collected from PEDV-infection piglets with or without *L. reuteri* transplantation. Statistical analysis was performed to quantify CA **(d)** and CDCA **(e)** content in fermentation broth of different treatment groups. **(f)** Agarose gel electrophoresis analysis of PCR-amplified BSHs genes. Lanes 1, 2, and 3 correspond to the 2000 bp DNA marker, PCR product amplified from MRS and *L. reuteri*, respectively. Data represent mean ± SD (one-way ANOVA). Significance: **P* < 0.05; ***P* < 0.01; ****P* <0.001; *****P* < 0.0001 **(d,e)**.

SUPPLEMENTARY FIGURE S8 Multiomics analysis reveals HDCA modulates NF-κB and ISG15 pathways against PEDV. Principal component analysis (PCA) of the transcriptomic **(a)** and proteomic **(b)** datasets. Venn diagram analysis of the transcriptomic **(c)** and proteomic **(d)** datasets. KEGG functional annotation analysis on the proteomic datasets. Differential expression analysis of the transcriptomic **(e)** and proteomic **(f)** datasets. **(g)** KEGG functional annotation analysis of proteomic datasets with significant differences. **(h)** KEGG pathway enrichment analysis of proteomic datasets with significant differences. **(i)** Cluster analysis of proteins related to Infectious disease: viral in KEGG Second Category. **(j)** Analysis of protein expression levels for key interferon-stimulated genes (ISGs) by heatmap.
