# Correction: *Lactobacillus reuteri*-derived HDCA suppresses PEDV replication while alleviating virus-triggered inflammation in piglets

**DOI:** 10.3389/fmicb.2025.1766552

**Published:** 2026-01-12

**Authors:** Zhibiao Bian, Qianqian Li, Hongchao Gou, Yan Li, Zhiyong Jiang, Pinpin Chu, Shaolun Zhai, Huahua Kang, Chunling Li, Guanghui Zhao

**Affiliations:** 1 College of Veterinary Medicine, Northwest A&F University, Yangling, China; 2 State Key Laboratory of Swine and Poultry Breeding Industry, Institute of Animal Health, Guangdong Academy of Agricultural Sciences, Guangzhou, China; 3 Guangdong Provincial Key Laboratory of Livestock Disease Prevention, Guangzhou, China; 4 Scientific Observation and Experiment Station of Veterinary Drugs and Diagnostic Techniques of Guangdong Province, Guangzhou, China

**Keywords:** *Lactobacillus reuteri*, HDCA, PEDV, gut microbiota, piglets

There was a mistake in the caption of Supplementary Figure S3 and S4 as published. Specifically, the figure intended to be Supplementary Figure S4 is listed as S3 in the legend, and Supplementary Figure S8 is listed as S4. The corrected caption of Supplementary Figure S4 and S8 appears below.

**Supplementary Figure S4**. Interaction analysis of *L. reuteri* and HDCA across experimental models. The Partial Least Squares Discriminant **(a)**, Venn diagram **(b)** and Differential expression **(c)** analysis was performed using untargeted metabolomics sequencing of jejunal contents collected from PEDV-infection piglets with or without *L. reuteri* transplantation. Statistical analysis was performed to quantify CA **(d)** and CDCA **(e)** content in fermentation broth of different treatment groups. **(f)** Agarose gel electrophoresis analysis of PCR-amplified BSHs genes. Lanes 1, 2, and 3 correspond to the 2,000 bp DNA marker, PCR product amplified from MRS and *L. reuteri*, respectively. Data represent mean ± SD (one-way ANOVA). Significance: *P < 0.05; **P < 0.01; ***P < 0.001; ****P < 0.0001 **(d, e)**.

**Supplementary Figure S8**. Multiomics analysis reveals HDCA modulates NF-κB and ISG15 pathways against PEDV. Principal component analysis (PCA) of the transcriptomic **(a)** and proteomic **(b)** datasets. Venn diagram analysis of the transcriptomic **(c)** and proteomic **(d)** datasets. KEGG functional annotation analysis on the proteomic datasets. Differential expression analysis of the transcriptomic **(e)** and proteomic **(f)** datasets. **(g)** KEGG functional annotation analysis of proteomic datasets with significant differences. **(h)** KEGG pathway enrichment analysis of proteomic datasets with significant differences. **(i)** Cluster analysis of proteins related to Infectious disease: viral in KEGG Second Category. **(j)** Analysis of protein expression levels for key interferon-stimulated genes (ISGs) by heatmap.

The original version of this article has been updated.

