# Supplementary figures and images for: Lactobacillus reuteri-derived HDCA suppresses PEDV replication while alleviating virus-triggered inflammation in piglets
Source: Front Microbiol. 2025 Nov 12;16:1669658. doi: 10.3389/fmicb.2025.1669658 (PMC12657016; doi:10.3389/fmicb.2025.1669658)

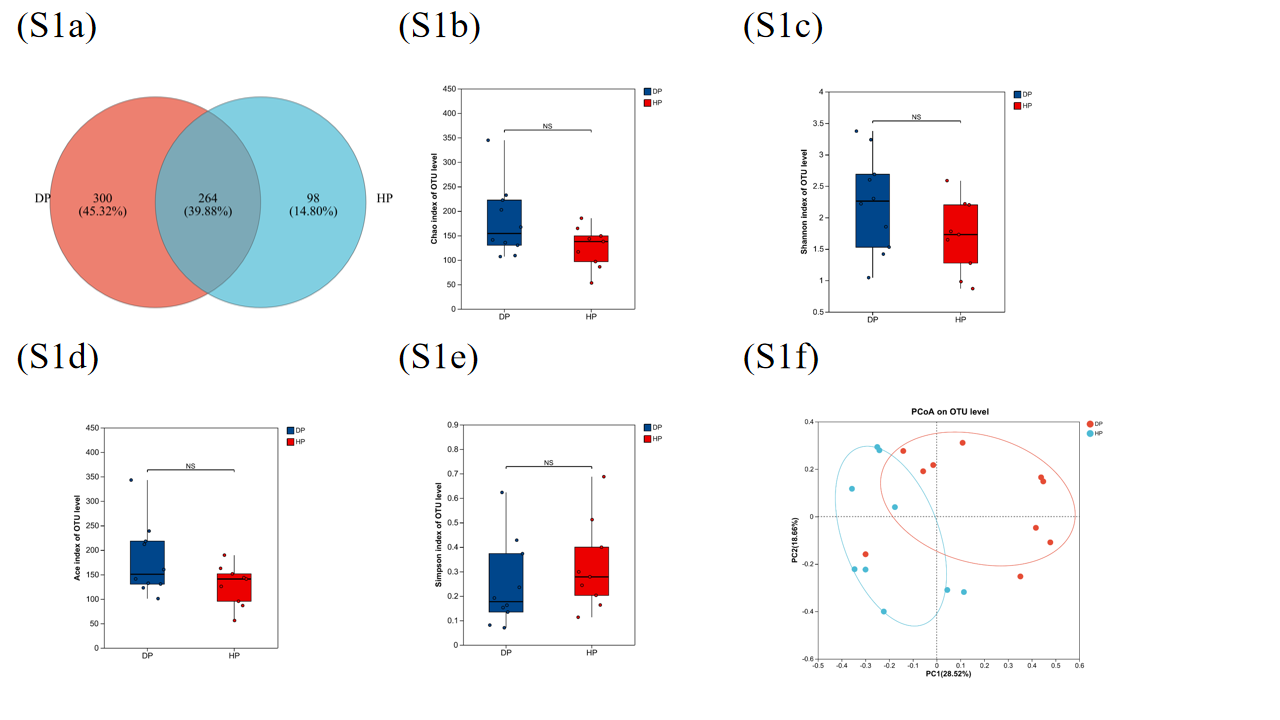

Supplement: SUPPLEMENTARY FIGURE S1 — Differences in the gut microbes of PEDV-infected diarrhea piglets (DP) and healthy control piglets (HP). (a) Comparative analysis of intestinal microbial OTUs of PEDV-infected diarrhea piglets (DP) and healthy control piglets (HP). Comparative analysis of Chao1 (b), Shannon (c), Ace (d), and Simpson (e) indices among different groups. (f) PCoA based on weighted bray curtis to identify microbial structural changes. [file Image_1.TIF]

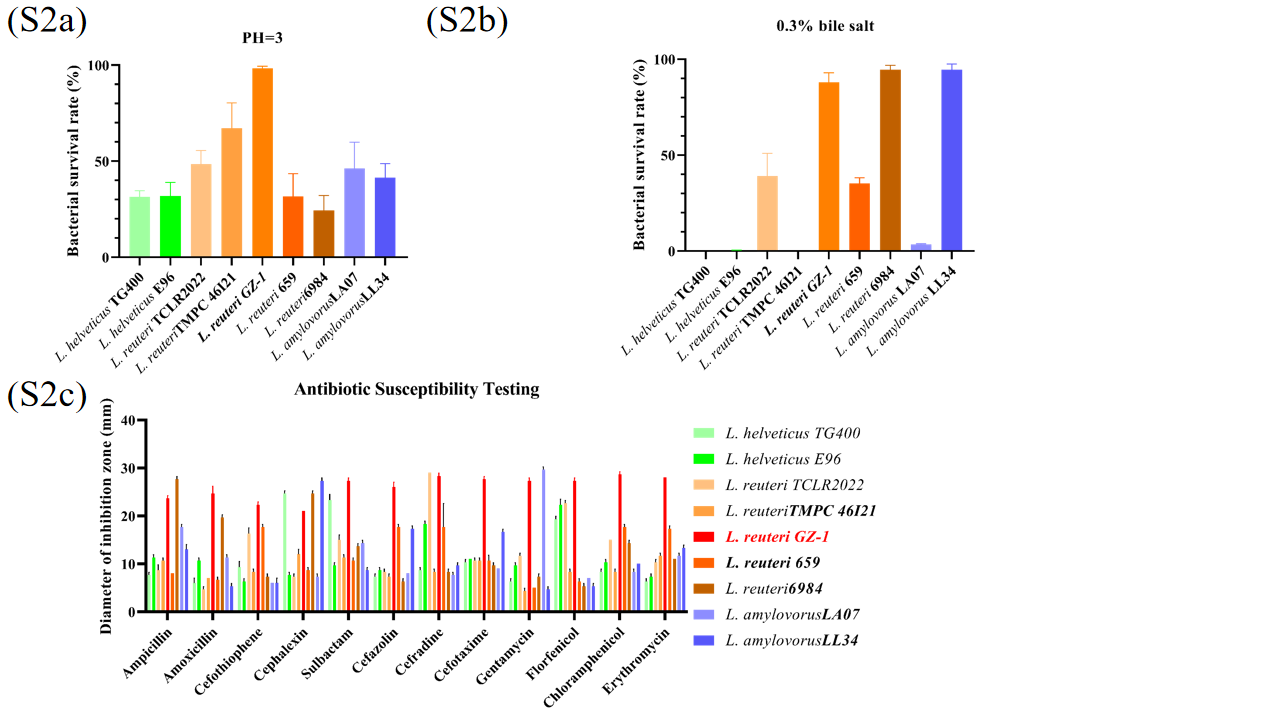

Supplement: SUPPLEMENTARY FIGURE S2 — Evaluation of L. reuteri GZ-1 properties and their impact on jejunal microbiota in piglets following transplantation. The acid resistance (a), bile salt resistance (b) and antibiotic resistance (c) of the strains were tested. Nonmetric multidimensional (d), principal co-ordinates analysis (e), community barplot analysis (f) and hierarchical clustering cluster analysis (g) of jejunal contents in each group. Data represent mean ± SD (a-c). [file Image_2.TIF]

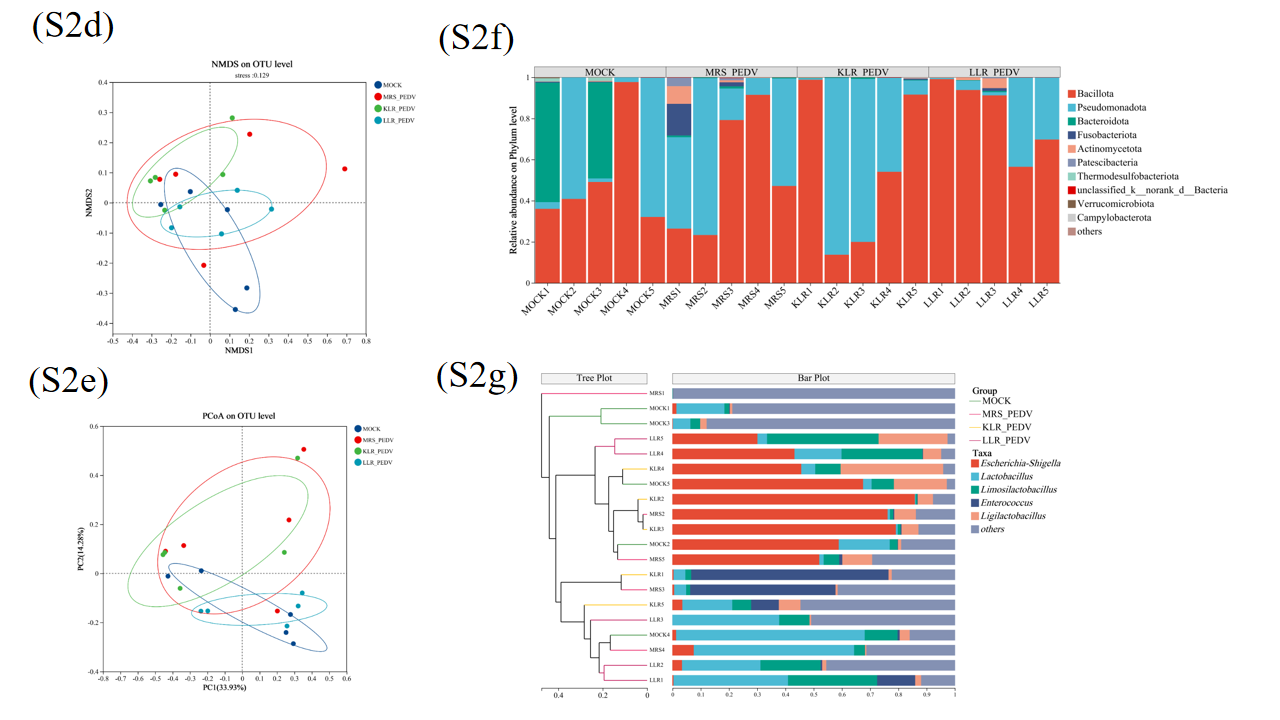

Supplement: SUPPLEMENTARY FIGURE S4 — Interaction analysis of L. reuteri and HDCA across experimental models. The Partial Least Squares Discriminant (a), Venn diagram (b) and Differential expression (c) analysis was performed using untargeted metabolomics sequencing of jejunal contents collected from PEDV-infection piglets with or without L. reuteri transplantation. Statistical analysis was performed to quantify CA (d) and CDCA (e) content in fermentation broth of different treatment groups. (f) Agarose gel electrophoresis analysis of PCR-amplified BSHs genes. Lanes 1, 2, and 3 correspond to the 2000 bp DNA marker, PCR product amplified from MRS and L. reuteri, respectively. Data represent mean ± SD (one-way ANOVA). Significance: *P < 0.05; **P < 0.01; ***P <0.001; ****P < 0.0001 (d,e). [file Image_3.TIF]

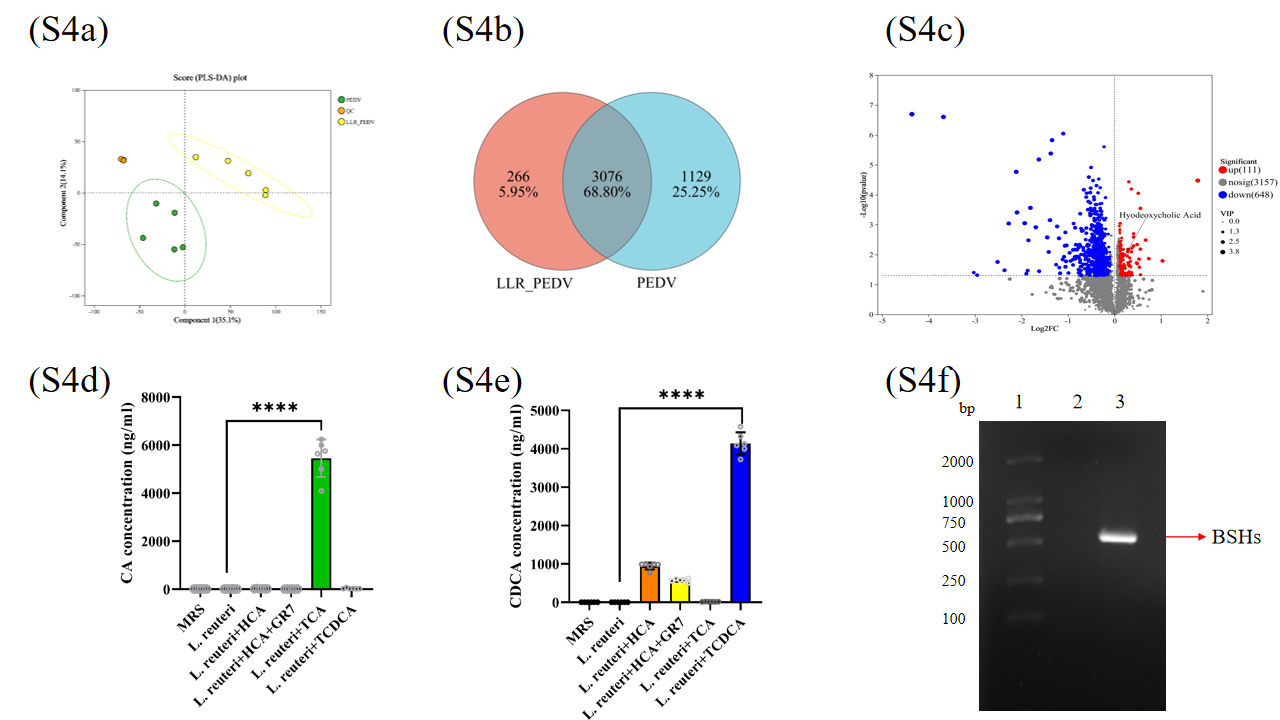

Supplement: SUPPLEMENTARY FIGURE S8 — Multiomics analysis reveals HDCA modulates NF-κB and ISG15 pathways against PEDV. Principal component analysis (PCA) of the transcriptomic (a) and proteomic (b) datasets. Venn diagram analysis of the transcriptomic (c) and proteomic (d) datasets. KEGG functional annotation analysis on the proteomic datasets. Differential expression analysis of the transcriptomic (e) and proteomic (f) datasets. (g) KEGG functional annotation analysis of proteomic datasets with significant differences. (h) KEGG pathway enrichment analysis of proteomic datasets with significant differences. (i) Cluster analysis of proteins related to Infectious disease: viral in KEGG Second Category. (j) Analysis of protein expression levels for key interferon-stimulated genes (ISGs) by heatmap. [file Image_4.TIF]

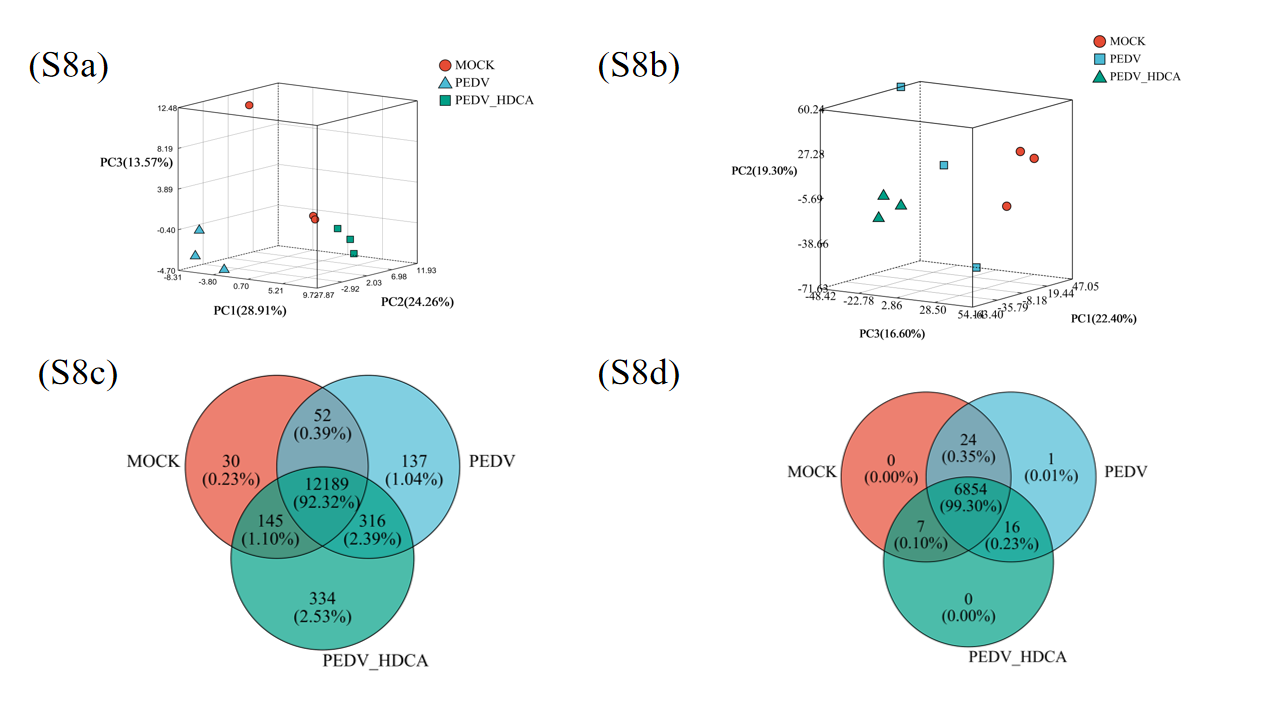

Supplement: Supplementary file 5 [file Image_5.TIF]

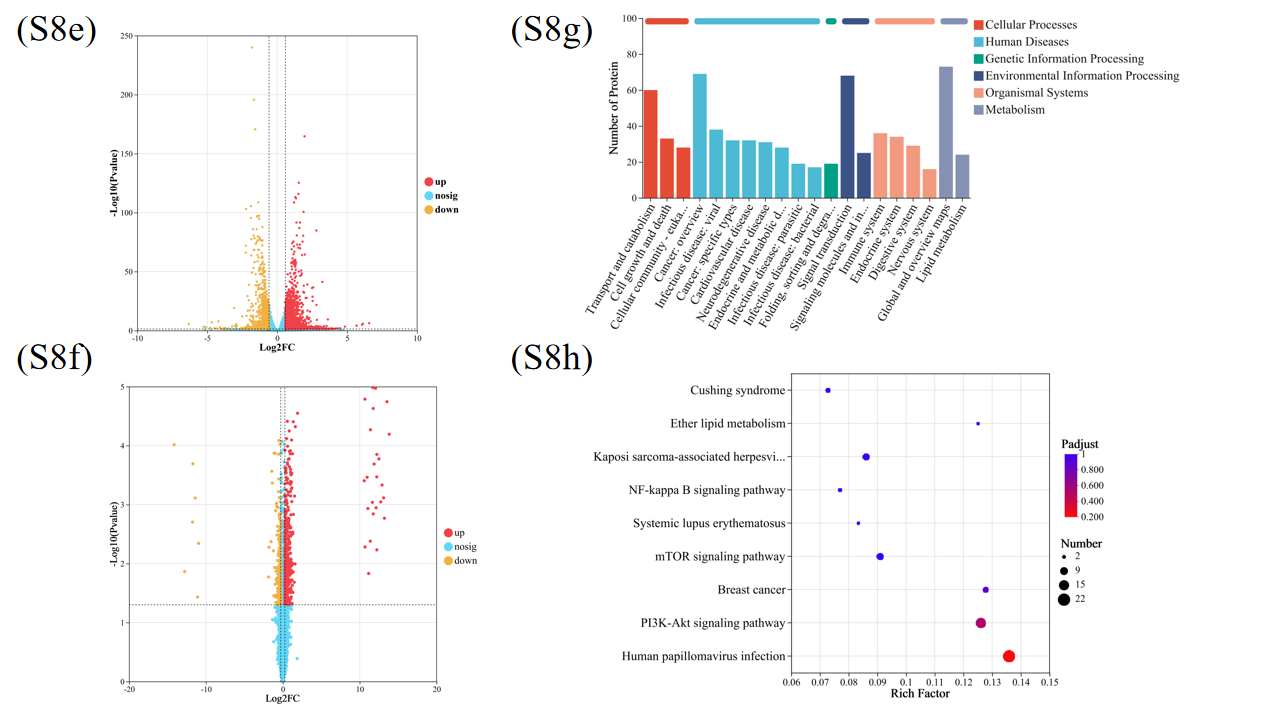

Supplement: Supplementary file 6 [file Image_6.TIF]

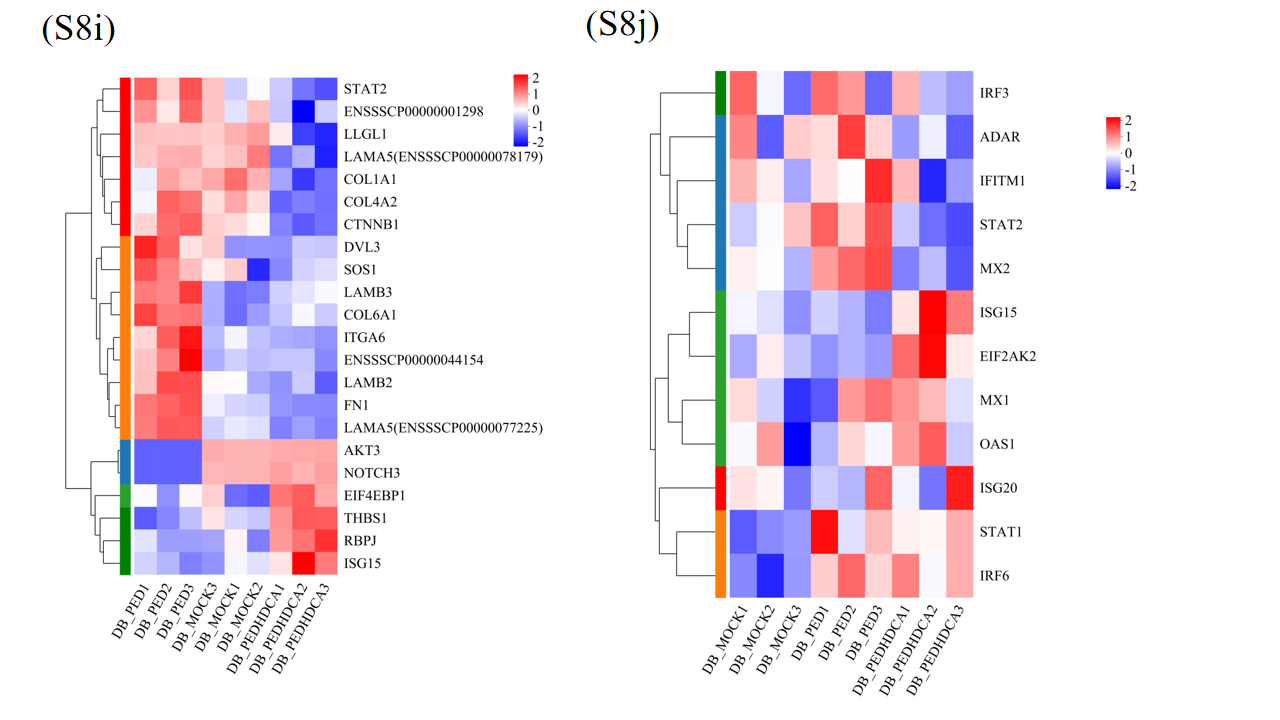

Supplement: Supplementary file 7 [file Image_7.TIF]
